# Identification of gene-sex hormone interactions associated with type 2 diabetes among men and women

**DOI:** 10.1371/journal.pgen.1011470

**Published:** 2025-09-02

**Authors:** Amonae Dabbs-Brown, Chang Liu, Qin Hui, Peter W.F. Wilson, Jin J. Zhou, Marta Gwinn, Yan V. Sun

**Affiliations:** 1 Department of Epidemiology, Emory University Rollins School of Public Health, Atlanta, Georgia, United States of America; 2 Division of Cardiology, Department of Medicine, Emory University School of Medicine, Atlanta, Georgia, United States of America; 3 VA Atlanta Health Care System, Decatur, Georgia, United States of America; 4 Department of Medicine, David Geffen School of Medicine, University of California, Los Angeles, California, United States of America; 5 Department of Biostatistics, Fielding School of Public Health, University of California, Los Angeles, California, United States of America; 6 Phoenix VA Health Care System, Phoenix, Arizona, United States of America; Vanderbilt University, UNITED STATES OF AMERICA

## Abstract

**Background and Objectives:**

Type 2 diabetes affects an increasing number of people worldwide. Although genome-wide association studies (GWAS) of type 2 diabetes have identified hundreds of loci, their interactions with other risk factors aren’t well understood. We investigated genetic interactions with three sex hormones (total testosterone, bioavailable testosterone, and sex hormone binding globulin (SHBG)) to identify additional type 2 diabetes-related loci that were undetected in traditional GWAS.

**Methods:**

The study population consisted of white European UK Biobank participants. Individuals with type 1 diabetes were excluded. We examined sex-stratified interactions of polygenic risk score (PRS) for type 2 diabetes with sex hormone levels. We analyzed sex-stratified, genome-wide SNP × sex hormone interactions, adjusting for age and the top ten principal ancestry components.

**Results:**

We found significant (P < 0.05) interactions for each of the sex hormones with PRS in both men and women, with the most significant being between SHBG and PRS in women (OR 0.88; 95% CI: 0.85-0.90; P = 1.09E-18). We identified 3 SNP × sex hormone interactions in men and 14 in women that achieved genome-wide significance (GWS; P < 5 × 10^-8^). Applying a 2-degree of freedom test, we identified GWS loci (10 in men and 23 in women) that were not GWS when testing marginal genetic effects alone.

**Conclusion:**

Including interaction terms in GWAS may identify additional risk loci and improve the understanding of genetic architecture for type 2 diabetes. Different genetic interactions with sex hormones in men and women emphasize the importance of sex-stratified analysis in sex differential diseases.

## Author summary

Type 2 diabetes is an increasingly common disease that is caused by both genetic and environmental factors. Though genome-wide association studies (GWAS) have been performed to identify several genes associated with type 2 diabetes, little is known about how an individual’s internal or external environment interacts with their genetics to affect disease outcomes. Here, we performed sex-stratified genome-wide interaction studies with 3 different sex-hormone interaction terms: testosterone, bio-available testosterone, and sex-hormone binding globulin. We identified 22 genomic risk loci for type 2 diabetes that were not reported in the most recent type 2 diabetes GWAS and were not significant using traditional GWAS. By including interaction terms in GWAS, we may be able to identify additional disease-related genes and gain a better understanding of the mechanisms behind complex diseases.

## Introduction

Type 2 diabetes is a major cause of blindness, kidney failure, heart attack, stroke, and lower limb amputation. It affects over 11% of the US population, and it is estimated that roughly 23% of individuals with diabetes (3.4% of all U.S. adults) are unaware that they have it [1,2]. According to the Centers for Disease Control National Diabetes Statistics Report, type 2 diabetes was the 8^th^ leading cause of death in the U.S. in 2021 with 31.1 deaths per 100,000 people [1]. Its global prevalence increased from 108 million in 1980–663 million in 2019 [2,3]. Risk factors associated with type 2 diabetes include tobacco smoking, high body mass index (BMI), low physical activity, high blood pressure, and greater age [1,4]. Epidemiological studies have demonstrated sex differences in type 2 diabetes in youth (<18 years old), with two-thirds of children and adolescents diagnosed with type 2 diabetes being girls [5]. However, among adults, prevalence of type 2 diabetes is higher in men [5]. In 2021, the International Diabetes Federation estimated that global diabetes prevalence in men was 10.8%, versus 10.2% in women [6]. In the U.S., the estimated diabetes prevalence from 2017-2020 was 14.2% in men and 12.4% in women [1].

Although both sexes produce the same sex hormones, their levels and bioavailability are vastly different. In men, the testes produce about 7,000 μg of testosterone per day and convert 0.25% into estradiol (E2), whereas in women, the ovaries produce about 300 μg of testosterone per day and convert 50% of it into E2 [7]. Testosterone also induces estrogen sulfotransferase (EST), which inactivates E2 [7]. The amounts of biologically available testosterone and E2 (i.e., testosterone and E2 available to target tissues) are modified by levels of albumin and sex-hormone binding globulin (SHBG). Albumin binds the sex hormones similarly; however, the affinity of SHBG for testosterone that is twice that for E2, and changes in SHBG levels greatly affect the amount of bioavailable testosterone (BAT).

Previous studies have observed that lower levels of testosterone confer greater risk of coronary events and type 2 diabetes in men but lower risk in women, while higher SHBG concentrations protect against coronary disease and type 2 diabetes in both sexes (although to a greater extent in women) [5,8]. A longitudinal study [9] including testosterone measurements for more than 70,000 men and 80,000 women found that men with serum testosterone <7 nmol/L had an adjusted type 2 diabetes incidence rate ratio (aIRR) of 2.71 when compared to men with testosterone ≥ 20 nmol/L; women with serum testosterone ≥ 3.5 nmol/L had an aIRR of 1.98 when compared to women with testosterone < 1.0 nmol/L. The same study reported that the risk of type 2 diabetes increased as the concentration of serum SHBG decreased to <40 in men and <50 nmol/L in women [9]. In a recent Mendelian randomization study, one standard deviation increase in genetically predicted testosterone levels was associated with higher risk of developing type 2 diabetes in women (odds ratio (OR) = 1.37) but a lower type 2 diabetes risk in men (OR = 0.86) [8]. Based on the evidence that testosterone levels affect the risk of type 2 diabetes differently in men and women, the Endocrine Society has recommended measuring free and total testosterone levels in all patients with type 2 diabetes [10].

Genetic factors also contribute to type 2 diabetes risk. The estimated heritability of type 2 diabetes ranges from 30 to 70% [11]. Genome-wide association studies (GWAS) have identified over 700 genetic loci associated with type 2 diabetes [12]. Genetic risk scores and polygenic risk scores (PRS) have been constructed to combine individual genetic effects; however, these genetic scores do not significantly enhance risk prediction over standard clinical risk factors [12,13]. Currently known risk loci explain only 10–20% of type 2 diabetes heritability, and the remaining 80–90% of heritability is likely due to variants that have yet to be detected due to their small effects and rare variants with low minor allele frequencies (MAF) [13]. Gene-gene and gene-environment/risk factor interactions can also contribute to this “missing heritability” [14].

Gene-environment interaction (G × E) analyses of type 2 diabetes-related outcomes have examined interactions with smoking, physical activity, and diet [15–19]. In 2020, Wu et al. [20] identified 5 loci associated with genotype-smoking interactions in type 2 diabetes risk. Kilpeläinen et al. reported 8 loci associated with gene-physical activity interactions and type 2 diabetes-related traits, including the conversion of impaired glucose intolerance to type 2 diabetes and blood lipid profile [17,18]. Gene-diet interaction studies have reported that 13–27% of phenotypic variance in fasting insulin and insulin resistance can be explained by gene-diet interactions [21,22]. Despite the well-established associations between sex hormones and type 2 diabetes [13–19], no published studies have investigated the interactions of genetic factors with sex hormone levels in relation to type 2 diabetes risk.

**Fig 1:**
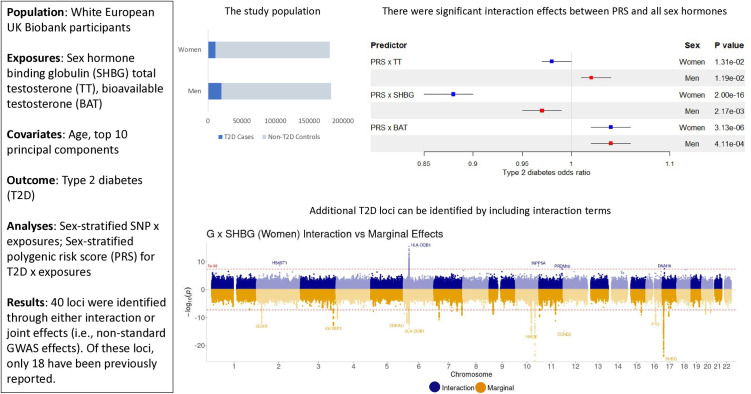
Graphical abstract.

Here, we analyze data from the UK Biobank (UKB) cohort to assess the effects of gene-sex hormone interactions on type 2 diabetes and to identify genetic variants that would not be found in type 2 diabetes GWAS examining only main genetic effects. A graphical representation of the study design and sampling can be found in Fig 1. We focused on measured total testosterone (TT) and SHBG and calculated BAT, which were available for most UKB participants.

## Results

### The study cohort

There were 484,127 UKB participants with basic phenotypic and genetic information available. Of these, 362,687 had TT measurements, 363,945 had SHBG measurements, and 294,727 had TT, SHBG, and albumin measurements allowing for the calculation of BAT. Characteristics of the TT sub-cohort, which is representative of all 3 sub-cohorts with sex hormone measures, are summarized in Table 1. The numbers of participants included in the genome-wide Gene Environment in Millions (GEM) analyses varied slightly, depending on the available data: 182,645 men and 180,773 women for TT, 169,872 men and 194,833 women for SHBG, and 150,798 men and 144,517 women for BAT.

**Table 1:** Characteristics of participants included in analysis of total testosterone x polygenic risk score interaction*.

	WOMEN			MEN		
	Controls	Cases	All	Controls	Cases	All
N	169388	11068	180456	162505	19726	182231
Age (years)	55.98 (7.96)	59.02 (7.4)	56.17 (7.96)	56.64 (8.12)	60.14 (6.82)	57.02 (8.06)
BMI (kg/m^2^)	26.78 (4.89)	32.23 (6.54)	27.11 (5.17)	27.44 (3.91)	31.21 (5.14)	27.84 (4.23)
Cholesterol (nmol/L)	5.94 (1.1)	5.34 (1.27)	5.9 (1.12)	5.59 (1.08)	4.77 (1.18)	5.5 (1.12)
PRS_T2D_	-0.19 (0.94)	0.46 (0.94)	-0.15 (0.96)	-0.21 (0.93)	0.43 (0.92)	-0.14 (0.95)
TT (nmol/L)	1.12 (0.63)	1.15 (0.68)	1.12 (0.63)	12.17 (3.68)	10.4 (3.57)	11.98 (3.71)
SHBG (nmol/L)	63.16 (30.09)	44.24 (26.86)	61.99 (30.25)	40.36 (16.63)	35.62 (16.74)	39.85 (16.71)
BAT (nmol/L)	0.35 (0.25)	0.46 (0.31)	0.36 (0.26)	5.27 (1.55)	4.76 (1.56)	5.21 (1.56)
CAD	9206 (5.43)	2404 (21.72)	11610 (6.43)	21663 (13.33)	6973 (35.35)	28636 (15.71)
Current smoker	15288 (9.03)	1336 (12.07)	16624 (9.21)	19361 (11.91)	2692 (13.65)	22053 (12.10)
Former smoker	53332 (31.49)	3918 (35.40)	57250 (31.73)	61619 (37.92)	9880 (50.09)	71499 (39.24)
Non-smoker	100205 (59.16)	5763 (52.07)	105968 (58.72)	81025 (49.86)	7024 (35.61)	88049 (48.32)
Post-Menopause†	102215 (60.34)	7354 (66.44)	109569 (60.72)	NA	NA	NA

*Analysis based on logistic regression model incorporating total testosterone x polygenic risk score as an interaction term, along with age and the first 10 principal components as covariates. The participants included in this model are representative of the participants included in all analyses reported in this study.

† Menopause status was available for only 153,947 women in this cohort.

Data are presented as count (percentage) or mean (standard deviation) as appropriate. Cases refer to participants with type 2 diabetes; controls refer to participants with no diabetes. N- number of participants, BMI – body mass index, PRS_T2D_ – polygenic risk score for type 2 diabetes, TT – total testosterone, SHBG – sex hormone binding globulin, BAT – bioavailable testosterone, CAD – coronary artery disease.

### PRS_T2D_ × sex hormone interactions in type 2 diabetes

Interactions between PRS_T2D_ and the sex hormones (TT, SHBG, and BAT) were examined separately in men and women (Table 2). The PRS_T2D_ was significantly associated with higher type 2 diabetes risk in all 6 models (ORs 1.85-2.34 per SD of PRS_T2D_). Higher TT and BAT levels were significantly associated with increased odds of type 2 diabetes in women (TT OR 1.08, C.I. 1.06-1.10; BAT OR 1.30, C.I. 1.27-1.32) and decreased odds in men (TT OR 0.61, C.I. 0.60-0.62; BAT OR 0.76, C.I. 0.75-0.78). Higher SHBG level was protective in both women (OR 0.45 C.I. 0.44-0.46) and men (OR 0.67, C.I. 0.66-0.69). The PRS_T2D_ × sex hormone interaction term was nominally significant (p < 0.05) in all 6 models, with the most significant interaction being between PRS_T2D_ and SHBG in women (OR 0.88, 95% C.I. 0.85-0.90, p < 2 × 10^-16^). A closer examination of the PRS_T2D_ × SHBG interaction in women showed that having both high SHBG and high PRS_T2D_ risk confers higher odds of developing type 2 diabetes than would be expected from additive or multiplicative effects and that an interaction may indeed be present (S1 Text, S2 Table).

**Table 2:** Logistic regression model results for sex hormone x PRS_T2D_ interaction analysis, stratified by sex.

Sex Hormone	Predictor	Women				Men			
		OR (95% C.I.)	P-value	N Cases	N Controls	OR (95% C.I.)	P-value	N Cases	N Controls
TT	PRS_T2D_	2.12 (2.05-2.19)	<1E-320	11,068	169,388	1.90 (1.80-2.01)	1.84E-109	19,726	162,505
	TT	1.08 (1.06-1.10)	3.52E-19			0.61 (0.60-0.62)	<1E-320		
	PRS_T2D_ x TT	0.98 (0.97-1.00)	0.01			1.02 (1.01-1.04)	0.01		
SHBG	PRS_T2D_	2.34 (2.23-2.46)	6.01E-265	12,502	181,955	2.15 (2.06-2.25)	1.21E-233	19,018	150,470
	SHBG	0.45 (0.44-0.46)	<1E-320			0.67 (0.66-0.69)	<1E-320		
	PRS_T2D_ x SHBG	0.88 (0.85-0.90)	1.09E-18			0.97 (0.95-0.99)	2.17E-03		
BAT	PRS_T2D_	1.91 (1.84-1.98)	1.04E-262	8,954	135,312	1.85 (1.73-1.97)	4.81E-79	16,681	133,600
	BAT	1.30 (1.27-1.32)	2.19E-174			0.76 (0.75-0.78)	1.09E-133		
	PRS_T2D_ x BAT	1.04 (1.02-1.06)	3.17E-06			1.04 (1.02-1.06)	4.11E-04		

TT- total testosterone, SHBG - sex hormone binding globulin, BAT- bioavailable testosterone, PRS_T2D_ – polygenic risk score for type 2 diabetes, T2D- type 2 diabetes, OR- odds ratio of T2D, C.I.- confidence interval. The interaction variable in the table is represented by PRS_T2D_ x hormone. The odds ratios shown are per increase in standard deviation. Age and principal components 1–10 were covariates in all models.

### Genome-wide SNP × sex hormone interactions in type 2 diabetes

GEM analysis of genome-wide significant (GWS; p < 5 × 10^-8^, 1-df) interactions identified fourteen relevant loci in women (4 interacting with SHBG, 10 with BAT), and two in men (1 interacting with TT and 1 with SHBG) (Table 3). Among women, 3 loci interacting with SHBG and 5 with BAT were also GWS in the analysis of joint (2 df) effects; the same was true for the locus interacting with TT among men. None of the loci identified via their interaction effects had marginal effects that were GWS.

**Table 3:** Genomic risk loci associated with type 2 diabetes through their interactions with sex hormones.

	SNPID	CHR	Position (hg19)	Nearest Gene	EA	NEA	EAF	P value	Beta (SE)
**Men**									
TT	rs118089799	9	80839160	GNAQ	A	G	9.1E-03	2.19E-09	-0.11 (0.02)
SHBG	rs738408	22	44324730	PNPLA3	T	C	0.23	4.09E-13	0.01 (0.001)
**Women**									
SHBG	rs72847791	2	129479940	AC012451.1	T	C	1.2E-02	2.40E-09	-0.02 (0.003)
SHBG	10:134562791:T:TCACG*	10	134562791	INPP5A	T	TCACG*	1.0E-02	3.46E-09	-0.02 (0.003)
SHBG	rs9804606	11	129889590	RP11-567M21.3	T	C	0.03	4.49E-08	-0.01 (0.003)
SHBG	rs140069179	17	11651941	DNAH9	C	A	0.05	3.83E-08	-0.01 (0.002)
BAT	rs34789050	3	51790029	IQCF6	A	G	0.05	1.02E-10	0.90 (0.14)
BAT	rs146037870	4	101861217	EMCN	T	C	0.03	4.15E-08	0.90 (0.16)
BAT	rs4476958	7	52954110	RP11-398K14.1	A	G	0.95	9.57E-09	-0.83 (0.15)
BAT	8:17032214:CTT:C	8	17032214	ZDHHC2	C	CTT	0.93	3.46E-11	-0.85 (0.13)
BAT	rs117878783	8	72988412	TRPA1	A	G	0.03	1.12E-08	1.05 (0.19)
BAT	rs80123595	10	119556155	RP11-355F22.1	T	C	0.03	1.50E-08	0.73 (0.13)
BAT	rs12268706	10	132418020	Y_RNA	C	T	0.06	9.93E-09	1.03 (0.18)
BAT	rs117530301	11	91819588	RPL7AP57	G	A	0.03	1.34E-08	1.13 (0.20)
BAT	rs12825501	12	30134559	LOC105369715	C	T	0.07	1.06E-08	0.67 (0.12)
BAT	rs17256233	14	61922461	PRKCH	T	C	0.10	2.32E-08	0.64 (0.11)

CHR- chromosome, SE – Standard error, EA – effect allele, NEA – non-effect allele, EAF – effect allele frequency, SHBG – sex hormone binding globulin, BAT – bioavailable testosterone.

* TCACGGTTGCAGCCACGCTTTGTTGGGGAGCAGCCAGCCTCAGGCCCTTCGC

More genomic loci with significant marginal or joint effects with TT, SHBG, or BAT were found among men than among women (S3 Table). Most loci with joint effects were also found to have marginal effects; those identified only by joint effects are described further in Table 4. There was no overlap between GWS loci identified for men and women within any hormone interaction analysis. Manhattan and QQ plots for each analysis are included in S1 Fig.

**Table 4:** Genomic risk loci associated with type 2 diabetes through their joint effects with sex hormones that do not overlap genomic risk loci identified through marginal effects.

	SNPID	CHR	Position (hg19)	Nearest Gene	EA	NEA	EAF	P value Joint	BETA (SE) Interaction	P value Interaction	BETA (SE) Genetic Main Effects	P value Genetic Main Effects
**Men**												
TT	rs141469161	7	2410296	EIF3B	C	T	0.99	3.29E-08	0.04 (0.02)	1.43E-02	0.29 (0.05)	6.00E-09
TT	rs118089799	9	80839160	GNAQ	G	A	0.99	1.60E-08	0.11 (0.02)	2.19E-09	0.04 (0.06)	0.476
TT	rs145010109	15	60620292	ANXA2	G	A	0.97	4.27E-08	0.05 (0.01)	3.01E-06	0.16 (0.03)	1.76E-06
TT	rs738409	22	44324727	PNPLA3	G	C	0.22	6.42E-09	0.02 (0.01)	4.48E-07	0.08 (0.02)	6.87E-08
SHBG	rs62580110	9	133865370	LAMC3	T	A	0.99	3.62E-10	0.02 (0.004)	7.39E-08	0.21 (0.05)	7.28E-05
SHBG	rs78328312	14	61842791	PRKCH	G	T	0.99	1.19E-09	0.01 (0.003)	4.46E-05	0.23 (0.04)	1.29E-07
SHBG	rs72720262	14	69682013	EXD2	G	A	0.33	6.42E-09	-0.003 (0.001)	2.05E-03	0.05 (0.01)	3.35E-05
SHBG	rs3747207	22	44324855	PNPLA3	A	G	0.21	3.86E-16	0.01 (0.001)	4.47E-13	0.09 (0.01)	1.63E-10
BAT	rs142442003	3	452463	CHL1	A	G	0.99	8.62E-09	0.15 (0.04)	6.10E-05	0.26 (0.05)	3.55E-07
BAT	rs271130	6	133246241	HMGB1P13	A	G	0.48	1.12E-08	-0.03 (0.01)	3.99E-03	0.05 (0.01)	6.04E-05
**Women**												
TT	rs1182395	7	157032924	UBE3C	T	G	0.65	4.98E-08	-0.04 (0.018)	3.82E-02	0.08 (0.01)	6.29E-08
SHBG	rs72847791	2	129479940	ISCA1P6	C	T	0.99	2.81E-09	0.02 (0.003)	2.40E-09	0.35 (0.07)	1.27E-06
SHBG	rs13266634	8	118184783	SLC30A8	C	T	0.69	1.37E-08	-0.003 (0.001)	4.04E-03	0.03 (0.02)	0.258
SHBG	rs112045042	9	71551592	PIP5K1B	C	T	0.99	1.05E-08	0.01 (0.003)	3.93E-07	0.34 (0.06)	3.58E-08
SHBG	10:134562791_ TCACGGTTGCAGCCACGCTTTGTT GGGGAGCAGCCAGCCTCAGGCCCTTCGCA_T	10	134562791	INPP5A	TCACGGTTGCAGCCACGCTTTGTT GGGGAGCAGCCAGCCTCAGGCCCTTCGCA	T	0.99	7.77E-09	0.02 (0.003)	3.46E-09	0.34 (0.08)	6.11E-06
SHBG	rs9804606	11	129889590	PRDM10	C	T	0.98	2.38E-09	0.01 (0.003)	4.49E-08	0.34 (0.06)	2.99E-09
SHBG	rs184922766	12	59633138	CCND2	G	C	0.99	4.48E-09	0.02 (0.003)	3.82E-07	0.42 (0.07)	5.47E-09
SHBG	rs71386896	16	85191033	CTC-786C10.1	G	A	0.98	2.69E-08	0.01 (0.003)	3.90E-07	0.31 (0.06)	2.97E-08
SHBG	rs117547596	22	34824038	SYN3	C	T	0.99	4.35E-08	-0.01 (0.004)	0.031038	0.13 (0.09)	0.143
BAT	rs114701560	2	30587510	Lnc-CAPN13–2	A	G	0.97	2.74E-08	-0.73 (0.16)	2.91E-06	0.15 (0.04)	3.57E-04
BAT	rs150147479	2	45057311	SIX3	A	G	0.98	1.76E-09	-0.68 (0.15)	3.27E-07	0.14 (0.04)	3.88E-04
BAT	rs13020842	2	122477115	NIFK	G	A	0.96	4.38E-08	-0.96 (0.19)	7.94E-06	0.17 (0.05)	1.23E-04
BAT	rs34789050	3	51790029	IQCF6	G	A	0.95	2.50E-10	-0.9 (0.14)	1.02E-10	0.05 (0.04)	0.138
BAT	rs149522	7	130449842	LOC105375508	T	C	0.40	3.28E-08	0.33 (0.08)	7.40E-05	0.06 (0.02)	1.84E-04
BAT	8:17032214_CTT_C	8	17032214	ZDHHC2	C	CTT	0.93	2.32E-10	-0.85 (0.13)	3.46E-11	0.03 (0.03)	0.360
BAT	rs147952627	10	119543721	LINC02674	C	G	0.97	3.56E-08	-0.73 (0.13)	2.00E-08	0.07 (0.03)	0.077
BAT	rs12268706	10	132418020	LOC100419870	C	T	0.06	9.03E-09	1.03 (0.18)	9.93E-09	0.09 (0.05)	0.027
BAT	rs61729748	11	5020933	OR51L1	A	T	0.98	2.28E-08	-0.87 (0.20)	1.30E-05	0.18 (0.05)	1.34E-04
BAT	rs12825501	12	30134559	LOC100422352	T	C	0.93	3.88E-08	-0.67 (0.18)	1.06E-08	0.04 (0.03)	0.145
BAT	rs118086999	15	38403041	LOC100507568	C	A	0.97	4.99E-08	-0.92 (0.18)	1.39E-07	0.10 (0.04)	0.021
BAT	rs551795351	17	76977993	CANT1	A	G	0.99	2.64E-08	-1.57 (0.33)	1.86E-06	0.20 (0.07)	5.28E-03
BAT	rs139416564	19	15172209	CASP14	T	C	0.99	4.21E-09	-1.83 (0.34)	9.80E-08	0.17 (0.07)	0.011
BAT	rs2545962	19	20591603	LOC100288623	A	C	0.99	4.22E-08	-1.66 (0.32)	3.34E-07	0.20 (0.07)	7.82E-03

Chr – chromosome, EA – effect allele, NEA – non-effect allele, EAF – effect allele frequency, TT – total testosterone, SHBG – sex hormone binding globulin, BAT – bioavailable testosterone.

### Sensitivity analyses

We performed two sensitivity studies in women to consider menopausal status. Firstly, menopausal status was added as a covariate in the GEM model, along with age at enrollment, PC1–10, and the G × SHBG or G × BAT interaction term (S1 Text). Participants without menopause information were excluded, reducing the sample size from 194,833–163,975 for SHBG and 144,517–122,581 for BAT. The interaction effects (beta coefficients) for the models with and without menopause as a covariate were highly correlated (r = 0.86 for SHBG and r = 0.93 for BAT) and the correlations between genome-wide significant SNPs was even higher (r = 0.98 for SHBG and r = 1.0 for BAT). These results indicated that the interactions we observed were not sensitive to a woman’s menopausal status (S2 Fig).

The second menopause sensitivity analysis was performed in only post-menopausal women. GEM models were built with age at enrollment, PC1–10, and the G × SHBG or G × BAT interaction terms as covariates (S1 Text). The sample sizes for the SHBG and BAT cohorts were 120,700, and 88,518, respectively. The interaction effects (beta coefficients) for the models in post-menopausal women vs all women were highly correlated (r = 0.76 for SHBG and r = 0.84 for BAT) and the correlations between genome-wide significant SNPs was even higher (r = 0.98 for SHBG and r = 1.0 for BAT). These results further indicated that the interactions we observed were not sensitive to a woman’s menopausal status (S3 Fig).

We performed a third sensitivity study to examine interaction effects in persons with incident type 2 diabetes. Incident cases were identified by comparing the date of initial assessment with the first date of type 2 diabetes diagnosis (S1 Text). Of the 50,647 type 2 diabetes cases in the UKB, 10,178 were incident. Beta coefficients for marginal and interaction effects in each G × hormone analysis for incident cases were compared to results from the corresponding analysis including both incident and prevalent type 2 diabetes. With the exception of SHBG interactions in men (r = 0.74), the correlations of genome-wide significant SNPs in all analyses were ≥ 0.94. Plots are shown in S4 Fig. The results of this analysis indicated that the interactions we observed were not sensitive to whether a person had incident or prevalent type 2 diabetes.

### Replication in African and South Asian cohorts

We attempted a small replication study in South Asian and African ancestry cohorts (S1 Text). In total, there were 7,502 participants in the South Asian cohort, 2,145 of whom had type 2 diabetes and 7,588 participants in the African cohort, 1,572 of whom had type 2 diabetes (S5 Table). The beta coefficients and p values for genomic risk loci identified via 1df and 2df interaction tests in the European cohort were then compared to their counterparts in the South Asian and African sub-cohorts. Generally, the coefficients showed very little correlation between ancestry groups (S6 Table), likely due to inaccurate estimates in the much smaller sample sizes with South Asian or African ancestry.

## Discussion

Observational studies of testosterone in relation to type 2 diabetes have found opposite effects in men and women: lower testosterone increases the risk of type 2 diabetes in men but decreases the risk in women. Biological processes underlying this phenomenon are not well understood. We undertook large-scale, sex-specific analyses of genetic interactions with measured testosterone and SHBG levels and calculated BAT in UK Biobank participants. Although interactions of all three biomarkers with a type 2 diabetes polygenic risk score were nominally statistically significant in both men and women, we found the best evidence for interaction with SBHG in women. We observed negative interaction between PRST2D and total testosterone (TT), which indicates an antagonistic interaction given the positive associations of both PRST2D and TT. Such statistical interaction suggests a potential shared etiology between PRST2D and TT which can mask out their effects on T2D. In a genome-wide analysis of SNP- sex hormone interactions, we identified 40 genomic risk loci for type 2 diabetes, of which only 18 overlap those reported in the most recent type 2 diabetes GWAS [23] (S4 Table). None of these SNPs had marginal effects that reached genome-wide significance.

Two protein coding genes, *PNPLA3* and *GNAQ,* mapped to the newly identified loci in men. Several studies have identified *PNPLA3* as being associated with testosterone and SHBG levels [8,24], and it has also been linked to type 2 diabetes [25]. The SNP rs738408 on *PNPLA3* is a synonymous variant that has been associated with Non-Alcoholic Fatty Liver Disease [26]. *GNAQ* has been associated primarily with body mass index (BMI) [27], which is also associated with both sex hormones and type 2 diabetes. In women, seven protein coding genes mapped to GWS interaction loci: *INPP5A, DNAH9, ZDHHC2, EMCN, PRKCH*, *TRPA1*, and *IQCF6*. GWAS have identified four of these (*INPP5A, ZDHHC2, IQCF6,* and *PRKCH*) to be associated with BMI [27,28] or obesity [29]. *INPP5A* has also been associated with of cardiovascular disease [28]. *EMCN* has been associated with left ventricular ejection fraction [30] and glomerular filtration rate [31]. *DNAH9* and *TRP1* have not been previously associated with any diabetes or sex-hormone related traits. Four of the 16 loci identified through interaction effects in either men or women -rs9804606, rs34789050, rs117530301, and rs12825501- are expression quantitative trait loci (eQTLs) which affect gene expression levels (S7 Table). The eQTL, rs34789050, is associated with genes TEX264, GRM2, and MANF and alter gene expression in several sex-associated tissues including vagina, testis, ovary, and mammary tissue.

These findings provide deeper insight into the modulating effects of sex and sex hormones on type 2 diabetes and emphasize the power of including both sex stratification and interaction terms in GWAS. Despite the known sex disparity for testosterone’s association with type 2 diabetes, it has not been examined in the context of gene-environment interactions. Previous GWAS have identified several loci showing sex-specific associations with type 2 diabetes-related traits. One 2012 study conducted by Morris et al. [32] found that sex stratification identified two additional loci associated with type 2 diabetes--*CCND2* in men and *GIPR* in women--that did not reach statistical significance when the sexes were combined. A 2014 study by Hara et al. [33] found evidence for 23 genes and regions displaying sexual dimorphism in associations with type 2 diabetes. In 2021, a study by Lagou et al. [34] reported 2 loci (*IRS1* and *ZNF12*) that display sexual dimorphism in effects on fasting insulin, a marker for diabetes.

Our analysis of genetic interactions with sex hormones in type 2 diabetes was limited in several important respects. First, hormone measurements for each participant were made at a single visit, without accounting for longitudinal variations. We were able to study only testosterone, sex hormone-binding globulin, and bioavailable testosterone because estradiol measurements were missing for most UK Biobank participants. G × Sex hormone analysis of sex chromosomal variants was not included, limited by data access and analysis issue. Finally, we were unable to access suitable data for replication analysis. We attempted to replicate our findings in Black and South Asian cohorts but were unsuccessful because of their small sample sizes. Future studies of sex hormones in type 2 diabetes would benefit from additional measurements in more diverse cohorts and could consider age stratification because hormone levels, particularly in women, vary greatly throughout the lifetime.

## Methods

### Ethics statement

The UKB received ethical approval from the National Information Governance Board for Health and Social Care and the National Health Service North West Centre for Research Ethics Committee (REC reference number 21/NW/0157) [23].

### Study cohort and phenotypes

The UKB includes about 500,000 individuals in the United Kingdom (UK) for whom detailed phenotypic and genotypic data were collected [35]. All participants provided written informed consent for their data to be used for health-related research at the time of recruitment.

Type 2 diabetes cases were identified using UKB hospital inpatient admission data and the self-reported data field “diabetes diagnosed by a doctor”. Hospital inpatient admission data were queried for type 2 diabetes diagnoses using International Classification of Diseases, 10th edition (ICD 10) codes E11-E14 and ICD-9 code 250. Participants with ICD-10 code E10 and ICD-9 codes 25001 and 25011 (type 1 diabetes) were excluded, as were any participants with self-reported type 1 diabetes. Participants with either prevalent or incident type 2 diabetes were considered cases for this analysis.

Plasma levels of total testosterone (TT), SHBG, and albumin measurements were attempted in all UKB participants at enrollment though some measurements fell outside the range of detection for their assay or did not pass quality control [24]. These individuals (69,024 missing TT; 69,544 missing SHBG; 65,150 missing albumin measurements) were excluded from the association analysis. Bio-available testosterone was calculated according to the Vermeulen equation [35–37] (S1 Text). We were unable to examine estradiol in this study because its measurement was missing for 92% of men and 79% of women.

### Genetic data

Genotyping was done using the Applied Biosystems UKB Axiom Array followed by data processing and quality control procedures considering batch, plate, and array effects, missing rate, Hardy-Weinberg Equilibrium, and sex mismatch, which are described in detail in the UKB genotyping documentation [38]. The dosages of genomic variants were then imputed by the UKB using the Haplotype Reference Consortium and UK10K + 1000 Genomes reference panels [38]. Only unrelated white Europeans with kinship < 0.0884 were included in our analyses. Ethnicity was self-reported during the patients’ initial Assessment Centre visit, and genetic data were used to estimate kinship coefficients, as described in the UKB genotyping documentation [38]. The first ten principal components (PC1–10) of ancestry were computed by the UKB, first on a subset of high-quality unrelated samples before the loadings were projected onto all samples [38], to account for population structure. Standard PRS for type 2 diabetes (PRS_T2D_) for the UKB participants (field ID 26285) were generated by the UKB from three GWAS datasets external to the UKB using genetic variants with INFO scores of imputation quality > 0.7 [39]. Briefly, PRS algorithms were built from trait-specific meta-analyses using a Bayesian approach, and per-individual PRS values were calculated as the genome-wide sum of the per-variant posterior effect size multiplied by allele dosage [39].

### G × Sex hormone interaction analysis

We used logistic regression to model the associations of PRS_T2D_ and each sex hormone level with type 2 diabetes status, stratified by sex. Age at enrollment and PC1–10 were included as covariates. We used this model to assess the marginal effects of PRS_T2D_ and sex hormones, as well as PRS_T2D_ × sex-hormone interaction terms. The GLM function of R version 4.0.3 was used to calculate raw and standardized estimates, which were then converted to odds ratios. In the standardized models, both the PRS_T2D_ and sex hormone variables were treated as continuous, and the odds ratios represent the change in type 2 diabetes risk per standard deviation increase in each respective variable.

The Gene-Environment interaction analysis for Millions of samples (GEM) approach enables GWAS that incorporates multiple exposures, controls for genotype-covariate interactions, and supports robust inference [40]. GEM considers a generalized linear model with interaction terms. In the present study, we implemented the following model: T2D ~ β_0_ + β_C_C + β_G_G + β_E_E + β_GE_(G × E) where T2D is the type 2 diabetes status, β_0_ is the intercept for the model; β_C_ and C represent the coefficients and values, respectively, for the covariates (age and PC1–10); β_G_ and G represent the coefficients and values for the genotype; β_E_ and E represent the coefficients and values for the exposure (sex hormone); and β_GE_ represents the coefficient for the interaction term G × E. Based on this equation, GEM calculates coefficient estimates and standard errors for interaction effects (G × E; 1 degree of freedom [1-df]) and genetic main effects (G), and also conducts joint tests (2 degrees of freedom [2-df]) of the genetic main and interaction effects (G + G × E) and reports the p values. Within the same analysis, GEM also considers a standard GWAS model with no interaction terms (T2D ~ β_0_ + β_C_C + β_G_G + β_E_E) and reports coefficient estimates, standard errors, and p values for the marginal genetic effects (G) of this model. It should be noted that within this manuscript, “marginal effects” will refer to genetic effects from a model with no interaction terms and “genetic main effects” will refer to genetic effects from a model with interaction terms. GEM version 1.4.2 was used to conduct sex-stratified gene-hormone interaction analysis with a type 2 diabetes outcome. Only common variants with MAF > 0.01 were included in the present study.

### Bioinformatic analysis

Functional Mapping and Annotation of Genome-Wide Association Studies (FUMA) is an online platform that can be used to annotate, prioritize, visualize, and interpret GWAS results [41]. The SNP2GENE function of FUMA version 1.5.1 was used under default settings to annotate the genomic loci identified from the robust marginal, joint, and interaction effects summary statistics output from GEM. For each analysis, FUMA identified genomic risk loci, lead SNPs, independently significant SNPs, and candidate GWAS-tagged SNPs. The definitions of these terms can be found in the FUMA documentation and are included in S1 Table [41]. Using the FUMA output, non-overlapping loci between the marginal (i.e., standard GWAS) and joint 2-df tests were identified based on chromosomal locations using the GenomicRanges package from Bioconductor [42].

## Supporting information

S1 Text Equations for the calculation of (A) free testosterone and (B) bioavailable testosterone [28,31]. Examination of the SHBG × PRS_T2D_ interaction in women. Menopause sensitivity analysis. Incident type 2 diabetes sensitivity analysis. Replication study in South Asian and African ancestry cohorts.
(DOCX)

S1 Table Functional Mapping and Annotation of Genome-Wide Association Studies (FUMA) definitions. (DOCX)

S2 Table SHBG x PRS_T2D_ interaction in women. (DOCX)

S3 Table The number of genomic risk loci identified by FUMA [28] for each hormone. (DOCX)

S4 Table Loci identified G x sex hormone interaction analysis that do not overlap those reported in the most recent large-scale type 2 diabetes GWAS [30]. (DOCX)

S1 Fig S1.1 A-C Fig. Manhattan and QQ Plots for the G x Total Testosterone analysis in men. **S1.2 A-C Fig.** Manhattan and QQ Plots for the G x Total Testosterone analysis in women. **S1.3 A-C Fig.** Manhattan and QQ Plots for the G x SHBG analysis in men. **S1.4 A-C Fig.** Manhattan and QQ Plots for the G x SHBG analysis in women. **S1.5 A-C Fig.** Manhattan and QQ Plots for the G x Bioavailable Testosterone analysis in men. **S1.6 A-C Fig.** Manhattan and QQ Plots for the G x Bioavailable Testosterone analysis in women.
(DOCX)

S2 Fig Correlation plots for the effects of adding menopause status as a covariate on GWS SNPs in BAT (A) and SHBG (B) interaction (1 df) analyses. (DOCX)

S3 Fig Correlation plots for the effects of excluding pre-menopausal women on GWS SNPs in BAT (A) and SHBG (B) interaction (1 df) analyses. (DOCX)

S4 Fig S4 A-F Fig. Correlation plots for incident type 2 diabetes sensitivity analyses. (DOCX)

S5 Table Summary statistics for the South Asian and African ancestry replication study cohorts. (DOCX)

S6 Table A comparison of genomic risk loci identified using interaction effects in Europeans to their counterparts in South Asian and African cohorts. (DOCX)

S7 Table Expression quantitative trait loci (eQTL) identified through interaction effects. (DOCX)

## References

[1] National Diabetes Statistics Report. Diabetes. 2022 [cited 2023 May 2]. https://www.cdc.gov/diabetes/data/statistics-report/index.html

[2] Diabetes. n.d. [cited 2023 May 2]. https://www.who.int/news-room/fact-sheets/detail/diabetes

[3] Ali MK, Pearson-Stuttard J, Selvin E, Gregg EW. Interpreting global trends in type 2 diabetes complications and mortality. Diabetologia. 2022;65(1):3–13. 34837505 10.1007/s00125-021-05585-2 PMC8660730

[4] National Institute of Diabetes and Digestive and Kidney Diseases. Risk Factors for Type 2 Diabetes. n.d. [cited 2023 May 2]. https://www.niddk.nih.gov/health-information/diabetes/overview/risk-factors-type-2-diabetes

[5] Huebschmann AG, Huxley RR, Kohrt WM, Zeitler P, Regensteiner JG, Reusch JEB. Sex differences in the burden of type 2 diabetes and cardiovascular risk across the life course. Diabetologia. 2019;62(10):1761–72. doi: 10.1007/s00125-019-4939-5 31451872 PMC7008947

[6] International Diabetes Federation. IDF Diabetes Atlas. 10th edn. Brussels, Belgium. 2021 [cited 2024 Jan 24]. https://diabetesatlas.org/

[7] Gambineri A, Pelusi C. Sex hormones, obesity and type 2 diabetes: is there a link?. Endocr Connect. 2018;8(1):R1-9. 10.1530/EC-18-0450 PMC6320346 30533003

[8] Ruth KS, Day FR, Tyrrell J, Thompson DJ, Wood AR, Mahajan A, et al. Using human genetics to understand the disease impacts of testosterone in men and women. Nat Med. 2020;26(2):252–8. doi: 10.1038/s41591-020-0751-5 32042192 PMC7025895

[9] O’Reilly MW, Glisic M, Kumarendran B, Subramanian A, Manolopoulos KN, Tahrani AA, et al. Serum testosterone, sex hormone-binding globulin and sex-specific risk of incident type 2 diabetes in a retrospective primary care cohort. Clin Endocrinol (Oxf). 2019;90(1):145–54. doi: 10.1111/cen.13862 30256433 PMC6334272

[10] Testosterone Therapy for Hypogonadism Guideline Resources. n.d. [cited 2023 May 2]. https://www.endocrine.org/clinical-practice-guidelines/testosterone-therapy

[11] Almgren P, Lehtovirta M, Isomaa B, Sarelin L, Taskinen MR, Lyssenko V, et al. Heritability and familiality of type 2 diabetes and related quantitative traits in the Botnia Study. Diabetologia. 2011;54(11):2811–9. doi: 10.1007/s00125-011-2267-5 21826484

[12] DeForest N, Majithia AR. Genetics of Type 2 Diabetes: Implications from Large-Scale Studies. Curr Diab Rep. 2022;22(5):227–35. doi: 10.1007/s11892-022-01462-3 35305202 PMC9072491

[13] Xue A, Wu Y, Zhu Z, Zhang F, Kemper KE, Zheng Z, et al. Genome-wide association analyses identify 143 risk variants and putative regulatory mechanisms for type 2 diabetes. Nat Commun. 2018;9(1):2941. doi: 10.1038/s41467-018-04951-w 30054458 PMC6063971

[14] Lamri A, De Paoli M, De Souza R, Werstuck G, Anand S, Pigeyre M. Insight into genetic, biological, and environmental determinants of sexual-dimorphism in type 2 diabetes and glucose-related traits. Front Cardiovasc Med. 2022;9:964743. doi: 10.3389/fcvm.2022.964743 36505380 PMC9729955

[15] Francis M, Li C, Sun Y, Zhou J, Li X, Brenna JT, et al. Genome-wide association study of fish oil supplementation on lipid traits in 81,246 individuals reveals new gene-diet interaction loci. PLoS Genet. 2021;17(3):e1009431. doi: 10.1371/journal.pgen.1009431 33760818 PMC8021161

[16] Hartiala JA, Hilser JR, Biswas S, Lusis AJ, Allayee H. Gene-Environment Interactions for Cardiovascular Disease. Curr Atheroscler Rep. 2021;23(12):75. doi: 10.1007/s11883-021-00974-9 34648097 PMC8903169

[17] Kilpeläinen TO, Lakka TA, Laaksonen DE, Laukkanen O, Lindström J, Eriksson JG, et al. Physical activity modifies the effect of SNPs in the SLC2A2 (GLUT2) and ABCC8 (SUR1) genes on the risk of developing type 2 diabetes. Physiol Genomics. 2007;31(2):264–72. doi: 10.1152/physiolgenomics.00036.2007 17636114

[18] Kilpeläinen TO, Bentley AR, Noordam R, Sung YJ, Schwander K, Winkler TW. Multi-ancestry study of blood lipid levels identifies four loci interacting with physical activity. Nat Commun. 2019;10(1):376. 30670697 10.1038/s41467-018-08008-w PMC6342931

[19] Lee YC, Lai CQ, Ordovas JM, Parnell LD. A database of gene-environment interactions pertaining to blood lipid traits, cardiovascular disease and type 2 diabetes. J Data Min Genomics Proteomics. 2011;2(1):106. 10.4172/2153-0602.1000106 PMC3275815 22328972

[20] Wu P, Rybin D, Bielak LF, Feitosa MF, Franceschini N, Li Y, et al. Smoking-by-genotype interaction in type 2 diabetes risk and fasting glucose. PLoS One. 2020;15(5):e0230815. doi: 10.1371/journal.pone.0230815 32379818 PMC7205201

[21] Zheng J-S, Lai C-Q, Parnell LD, Lee Y-C, Shen J, Smith CE, et al. Genome-wide interaction of genotype by erythrocyte n-3 fatty acids contributes to phenotypic variance of diabetes-related traits. BMC Genomics. 2014;15(1):781. doi: 10.1186/1471-2164-15-781 25213455 PMC4168207

[22] Zheng J-S, Arnett DK, Lee Y-C, Shen J, Parnell LD, Smith CE, et al. Genome-wide contribution of genotype by environment interaction to variation of diabetes-related traits. PLoS One. 2013;8(10):e77442. doi: 10.1371/journal.pone.0077442 24204828 PMC3810463

[23] Suzuki K, Hatzikotoulas K, Southam L, Taylor HJ, Yin X, Lorenz KM, et al. Genetic drivers of heterogeneity in type 2 diabetes pathophysiology. Nature. 2024;627(8003):347–57. doi: 10.1038/s41586-024-07019-6 38374256 PMC10937372

[24] Fantus RJ, Na R, Wei J, Shi Z, Resurreccion WK, Halpern JA. Genetic Susceptibility for Low Testosterone in Men and Its Implications in Biology and Screening: Data from the UK Biobank. Eur Urol Open Sci. 2021;29:36–46. 34337532 10.1016/j.euros.2021.04.010 PMC8317803

[25] Mahajan A, Wessel J, Willems SM, Zhao W, Robertson NR, Chu AY, et al. Refining the accuracy of validated target identification through coding variant fine-mapping in type 2 diabetes. Nat Genet. 2018;50(4):559–71. doi: 10.1038/s41588-018-0084-1 29632382 PMC5898373

[26] Najafi M, Rafiei A, Ghaemi A, Hosseini V. Association between *rs738408*, *rs738409* and *rs139051* polymorphisms in PNPLA3 gene and non-alcoholic fatty liver disease. Gene Rep. 2022;26:101472.

[27] Huang J, Huffman JE, Huang Y, Do Valle Í, Assimes TL, Raghavan S, et al. Genomics and phenomics of body mass index reveals a complex disease network. Nat Commun. 2022;13(1):7973. doi: 10.1038/s41467-022-35553-2 36581621 PMC9798356

[28] Kichaev G, Bhatia G, Loh PR, Gazal S, Burch K, Freund MK. Leveraging Polygenic Functional Enrichment to Improve GWAS Power. Am J Hum Genet. 2019;104(1):65–75. 30595370 10.1016/j.ajhg.2018.11.008 PMC6323418

[29] Wheeler E, Huang N, Bochukova EG, Keogh JM, Lindsay S, Garg S, et al. Genome-wide SNP and CNV analysis identifies common and low-frequency variants associated with severe early-onset obesity. Nat Genet. 2013;45(5):513–7. doi: 10.1038/ng.2607 23563609 PMC4106235

[30] Deng X, Sabino EC, Cunha-Neto E, Ribeiro AL, Ianni B, Mady C, et al. Genome wide association study (GWAS) of Chagas cardiomyopathy in Trypanosoma cruzi seropositive subjects. PLoS One. 2013;8(11):e79629. doi: 10.1371/journal.pone.0079629 24324551 PMC3854669

[31] Liu H, Doke T, Guo D, Sheng X, Ma Z, Park J, et al. Epigenomic and transcriptomic analyses define core cell types, genes and targetable mechanisms for kidney disease. Nat Genet. 2022;54(7):950–62. doi: 10.1038/s41588-022-01097-w 35710981 PMC11626562

[32] Morris AP, Voight BF, Teslovich TM, Ferreira T, Segrè AV, Steinthorsdottir V, et al. Large-scale association analysis provides insights into the genetic architecture and pathophysiology of type 2 diabetes. Nat Genet. 2012;44(9):981–90. doi: 10.1038/ng.2383 22885922 PMC3442244

[33] Hara K, Fujita H, Johnson TA, Yamauchi T, Yasuda K, Horikoshi M, et al. Genome-wide association study identifies three novel loci for type 2 diabetes. Hum Mol Genet. 2014;23(1):239–46. doi: 10.1093/hmg/ddt399 23945395

[34] Lagou V, Mägi R, Hottenga JJ, Grallert H, Perry JRB, Bouatia-Naji N, et al. Sex-dimorphic genetic effects and novel loci for fasting glucose and insulin variability. Nature Communications. 2021;12:24. doi: 10.1038/s41467-020-20112-5 PMC7785747 33402679

[35] Conroy MC, Lacey B, Bešević J, Omiyale W, Feng Q, Effingham M, et al. UK Biobank: a globally important resource for cancer research. Br J Cancer. 2023;128(4):519–27. doi: 10.1038/s41416-022-02053-5 36402876 PMC9938115

[36] Watts EL, Perez-Cornago A, Knuppel A, Tsilidis KK, Key TJ, Travis RC. Prospective analyses of testosterone and sex hormone-binding globulin with the risk of 19 types of cancer in men and postmenopausal women in UK Biobank. Int J Cancer. 2021;149(3):573–84. 33720423 10.1002/ijc.33555

[37] Vermeulen A, Verdonck L, Kaufman JM. A critical evaluation of simple methods for the estimation of free testosterone in serum. J Clin Endocrinol Metab. 1999;84(10):3666–72. doi: 10.1210/jcem.84.10.6079 10523012

[38] Bycroft C, Freeman C, Petkova D, Band G, Elliott LT, Sharp K, et al. The UK Biobank resource with deep phenotyping and genomic data. Nature. 2018;562(7726):203–9. doi: 10.1038/s41586-018-0579-z 30305743 PMC6786975

[39] UK Biobank release and systematic evaluation of optimised polygenic risk scores for 53 diseases and quantitative traits. medRxiv. n.d. [cited 024 June 24]. https://www.medrxiv.org/content/10.1101/2022.06.16.22276246v1

[40] Westerman KE, Pham DT, Hong L, Chen Y, Sevilla-González M, Sung YJ. GEM: scalable and flexible gene–environment interaction analysis in millions of samples. Bioinformatics. 2021;37(20):3514–20. doi: 10.1093/bioinformatics/btab204 34695175 PMC8545347

[41] Watanabe K, Taskesen E, van Bochoven A, Posthuma D. Functional mapping and annotation of genetic associations with FUMA. Nat Commun. 2017;8(1):1826. 29184056 10.1038/s41467-017-01261-5 PMC5705698

[42] Lawrence M, Huber W, Pagès H, Aboyoun P, Carlson M, Gentleman R, et al. Software for computing and annotating genomic ranges. PLoS Comput Biol. 2013;9(8):e1003118. doi: 10.1371/journal.pcbi.1003118 23950696 PMC3738458

