# Supplementary material for: Identification of gene-sex hormone interactions associated with type 2 diabetes among men and women
Source: PLoS Genet. 2025 Sep 2;21(9):e1011470. doi: 10.1371/journal.pgen.1011470 (PMC12419643; doi:10.1371/journal.pgen.1011470)
Supplement: S1 Text — Examination of the SHBG × PRST2D interaction in women. Menopause sensitivity analysis. Incident type 2 diabetes sensitivity analysis. Replication study in South Asian and African ancestry cohorts. (DOCX) [file pgen.1011470.s001.docx]

# Supplementary Methods

## Equations for the calculation of (A) free testosterone and (B) bioavailable testosterone (28,31)

1. $[FT]=\frac{-b+\sqrt{b^{2}-4a*[TT]}}{2a}$   , (mol/L)

a = Kat + Kt+ (Kat * Kt) ([SHBG] + [albumin] − [TT])

b = 1 + Kt * [SHBG] + Kat *[albumin] − (Kat + Kt) *[TT]

- Kat: Association constant of testosterone for albumin = 3.6 x 10^4^ L/mol
- Kt: Association constant of testosterone for SHBG = 10 x 10^8^ L/mol
- [TT]: Serum total testosterone concentration in mol/L
- [SHBG]: Sex hormone binding globulin concentration in mol/L.
- [albumin]: albumin concentration in mol/L
- [FT]: Serum free testosterone concentration in mol/L

1. [BT]=[𝐹𝑇]+(𝐾𝑎∗[albumin]∗[𝐹𝑇])

- Kat: Association constant of testosterone for albumin = 3.6 x 10^4^ L/mol
- [albumin]: Concentration of albumin (mol/L)
- [FT]: Free testosterone concentration (mol/L)
- [BT]: Bioavailable testosterone concentration (mol/L)

## Examination of the SHBG×PRS_T2D_ interaction in women

To better illustrate how SHBG and PRS_T2D_ levels interact to affect type 2 diabetes odds in women, female participants were labeled according to PRS_T2D_ and SHBG risks: low (< median) and high (≥ median). These labels were used to divide the cohort into four risk groups: low PRS risk and low SHBG risk (LL); low PRS risk and high SHBG risk (LH); high PRS risk and low SHBG risk (HL); high PRS risk and high SHBG risk (HH). Logistic regression was used to estimate the standardized ORs of type 2 diabetes for each risk group compared to the LL group (Supplementary Table S2). The LH and HL groups had ORs (95% C.I.) of 2.95 (2.73-3.19) and 2.63 (2.42-2.85), respectively, while the HH group had an OR (95% C.I.) of 9.07 (8.47-9.73). The OR of the HH group is higher than one would expect from simple additive or multiplicative effects, indicating that an interaction between SHBG and PRS_T2D_ may be present.

## Menopause sensitivity analyses

Sensitivity studies were conducted to test the effects of menopause status on the interaction between genotype and SHBG and bioavailable testosterone levels in women. Menopause status was collected via patient questionnaire in the initial assessment center visit (23); about 70% of female UKB participants have experienced menopause. In one sensitivity analysis, patients' menopause status was added as a covariate in the GEM model along with age at enrollment, PC1-10, and the G x SHBG (BAT) interaction term. Correlation plots comparing the interaction beta coefficients for the GEM model with and without menopause status as a covariate are shown in Supplementary Figure S6. In the second menopause sensitivity analysis, GEM models were built for only post-menopausal women. Age at enrollment, PC1-10, and the G x SHBG (BAT) interaction terms were included as covariates. Correlation plots comparing the interaction beta coefficients for models in all women vs post-menopausal women are shown in Supplementary Figure S7.

## Incident type 2 diabetes sensitivity analysis

In the main text, participants with type 2 diabetes at the initial assessment (prevalent type 2 diabetes) and those who developed type 2 diabetes after the initial assessment (incident type 2 diabetes) were not separated during interaction analyses. This was done with the assumption that development of type 2 diabetes does not alter hormone levels. To be sure, GEM (33) was re-run under the same methods as the main text using only participants with incident type 2 diabetes as cases and participants with no type 2 diabetes as controls. Cases were identified by comparing the date of initial assessment with the first date of type 2 diabetes diagnosis. Of the 50,647 type 2 diabetes cases in the UKB, 10,178 were incident type 2 diabetes. Beta coefficients for marginal and interaction effects in each G × hormone analysis were compared to their counterpart using the combined type 2 diabetes outcome. With the exception of SHBG interaction effects in men (r = 0.74), the correlation of genome-wide significant SNPs in all analyses was ≥ 0.94. Plots are shown in Supplementary Figures S8.

## Replication study in South Asian and African ancestry cohorts

The GEM analysis was replicated in participants with South Asian and African ancestries. As with the European study, self-reported ethnicities were used and individuals with kinship > 0.884 were excluded. In total, there were 7,502 participants in the South Asian cohort, 2,145 of whom had type 2 diabetes and 7,588 participants in the African cohort, 1,572 of whom had type 2 diabetes (S5 Table). Sex stratified gene x sex hormone analysis was run using GEM under the same methods as the main text. The beta coefficients for genomic risk loci identified via interaction effects in the European cohort were then compared to their counterparts in the South Asian and African cohorts. Generally, the coefficients showed very little correlation between ancestry groups (S6 Table).
