# Supplementary material for: Identification of gene-sex hormone interactions associated with type 2 diabetes among men and women
Source: PLoS Genet. 2025 Sep 2;21(9):e1011470. doi: 10.1371/journal.pgen.1011470 (PMC12419643; doi:10.1371/journal.pgen.1011470)
Supplement: S1 Table — (DOCX) [file pgen.1011470.s002.docx]

**S1 Table**: Functional Mapping and Annotation of Genome-Wide Association Studies (FUMA) definitions.

| Independently significant SNPs | *P* < 5 × 10−8 and independent from each other at *r*2 < 0.6 |
| --- | --- |
| Candidate SNPs | SNPs with *r*2 ≥ 0.6 with one of the independent significant SNPs, with a minor allele frequency ≥0.01 |
| Lead SNPs | Independently significant SNPs with *r*2 < 0.1 |
| Genomic risk loci | Identified by merging lead SNPs within a 250 kb window and all SNPs in linkage disequilibrium of *r*2 ≥ 0.6 with one of the independent SNPs. |
