# Supplementary material for: Identification of gene-sex hormone interactions associated with type 2 diabetes among men and women
Source: PLoS Genet. 2025 Sep 2;21(9):e1011470. doi: 10.1371/journal.pgen.1011470 (PMC12419643; doi:10.1371/journal.pgen.1011470)
Supplement: S2 Table — (DOCX) [file pgen.1011470.s003.docx]

**S2 Table**: SHBG x PRS_T2D_ interaction in women

| **PRS_T2D_ Risk** | **SHBG Risk** | **Number of Cases** | **Number of Controls** | **OR (95% CI)** | **P value** |
| --- | --- | --- | --- | --- | --- |
| Low | Low | 2,223 | 41,388 | Reference | Reference |
| Low | High | 921 | 52,697 | 2.95 (2.73-3.19) | < 2×10^-16^ |
| High | Low | 7,468 | 46,155 | 2.63 (2.42-2.85) | < 2×10^-16^ |
| High | High | 1,890 | 41,715 | 9.07 (8.47-9.73) | < 2×10^-16^ |

The table above shows odds ratios for type 2 diabetes dependent on high or low SHBG and PRS_T2D_ risk groups. High and low PRS_T2D_ risk are PRS_T2D_ values above and below the median, respectively. As SHBG is protective, high and low SHBG risk are SHBG concentrations below and above the median, respectively. ORs are compared to low PRS_T2D_ and low SHBG risk groups. SHBG - sex hormone binding globulin, PRS_T2D_ – polygenic risk score for type 2 diabetes, T2D- type 2 diabetes, OR – odds ratio, CI – confidence interval. The odds ratios shown are per increase in standard deviation. Age and principal components 1-10 were covariates.
