# Supplementary material for: Identification of gene-sex hormone interactions associated with type 2 diabetes among men and women
Source: PLoS Genet. 2025 Sep 2;21(9):e1011470. doi: 10.1371/journal.pgen.1011470 (PMC12419643; doi:10.1371/journal.pgen.1011470)
Supplement: S3 Table — (DOCX) [file pgen.1011470.s004.docx]

**S3 Table:** The number of genomic risk loci identified by FUMA (28) for each hormone and effect.

|  |  | **Women** | **Men** |
| --- | --- | --- | --- |
| **Hormone** | **Effect** | **N Genomic Risk Loci** | **N Genomic Risk Loci** |
| TT | Interaction | 0 | 1 |
| TT | Marginal | 21 | 45 |
| TT | Joint | 18 | 39 |
| TT | Joint, not marginal | 1 | 4 |
| SHBG | Interaction | 4 | 1 |
| SHBG | Marginal | 22 | 40 |
| SHBG | Joint | 28 | 38 |
| SBHG | Joint, not marginal | 8 | 4 |
| BAT | Interaction | 10 | 0 |
| BAT | Marginal | 17 | 34 |
| BAT | Joint | 26 | 28 |
| BAT | Joint, not marginal | 14 | 2 |

TT- total testosterone, SHBG- sex hormone binding globulin, BAT- bioavailable testosterone, N- count, FUMA- Functional mapping and annotation of genetic associations. “Joint, not marginal” refers to genome wide significant loci identified through the joint (2-degree of freedom) test that do not overlap genome wide significant loci identified through the marginal test.
