# Supplementary material for: Identification of gene-sex hormone interactions associated with type 2 diabetes among men and women
Source: PLoS Genet. 2025 Sep 2;21(9):e1011470. doi: 10.1371/journal.pgen.1011470 (PMC12419643; doi:10.1371/journal.pgen.1011470)
Supplement: S4 Table — (DOCX) [file pgen.1011470.s005.docx]

**S4 Table:** Loci identified G x sex hormone interaction analysis that do not overlap those reported in the most recent large-scale type 2 diabetes GWAS (30).

| **Index SNP ID** | **CHR** | **Start** | **End** | **Sex Hormone** |
| --- | --- | --- | --- | --- |
| rs114701560 | 2 | 30570440 | 30631507 | BAT |
| rs13020842 | 2 | 122119197 | 122492003 | BAT |
| rs72847791 | 2 | 129479940 | 129479940 | SHBG |
| rs142442003 | 3 | 367094 | 452463 | BAT |
| rs271130 | 6 | 133236406 | 133267403 | BAT |
| rs4476958 | 7 | 52906693 | 52987534 | BAT |
| rs141469161 | 7 | 2404539 | 2435018 | TT |
| 8:17032214_CTT_C | 8 | 17032214 | 17032214 | BAT |
| rs112045042 | 9 | 71551592 | 71551592 | SHBG |
| rs12268706 | 10 | 132412890 | 132427688 | BAT |
| 10:134562791_TCA*_T | 10 | 134340391 | 134562791 | SHBG |
| rs117530301 | 11 | 91819588 | 91935098 | BAT |
| rs61729748 | 11 | 5020933 | 5203863 | BAT |
| rs9804606 | 11 | 129858556 | 129889590 | SHBG |
| rs12825501 | 12 | 30134559 | 30134559 | BAT |
| rs184922766 | 12 | 59624693 | 59647968 | SHBG |
| rs17256233 | 14 | 61911178 | 61938155 | BAT |
| rs78328312 | 14 | 61817119 | 61842791 | SHBG |
| rs145010109 | 15 | 60519507 | 60620292 | TT |
| rs140069179 | 17 | 11550355 | 11663172 | SHBG |
| rs139416564 | 19 | 15115075 | 15172209 | BAT |
| rs117547596 | 22 | 34671869 | 34962560 | SHBG |

*CHR- chromosome, TT – total testosterone, SHBG – sex hormone binding globulin, BAT – bioavailable testosterone*
