# Supplementary material for: Identification of gene-sex hormone interactions associated with type 2 diabetes among men and women
Source: PLoS Genet. 2025 Sep 2;21(9):e1011470. doi: 10.1371/journal.pgen.1011470 (PMC12419643; doi:10.1371/journal.pgen.1011470)

**S1.1 A-C Fig:** Manhattan and QQ Plots for the G x Total Testosterone analysis in men. GWAS inflation factors for interaction, joint, and marginal effects were 1.02, 1.11, and 1.12, respectively.

**A**

**
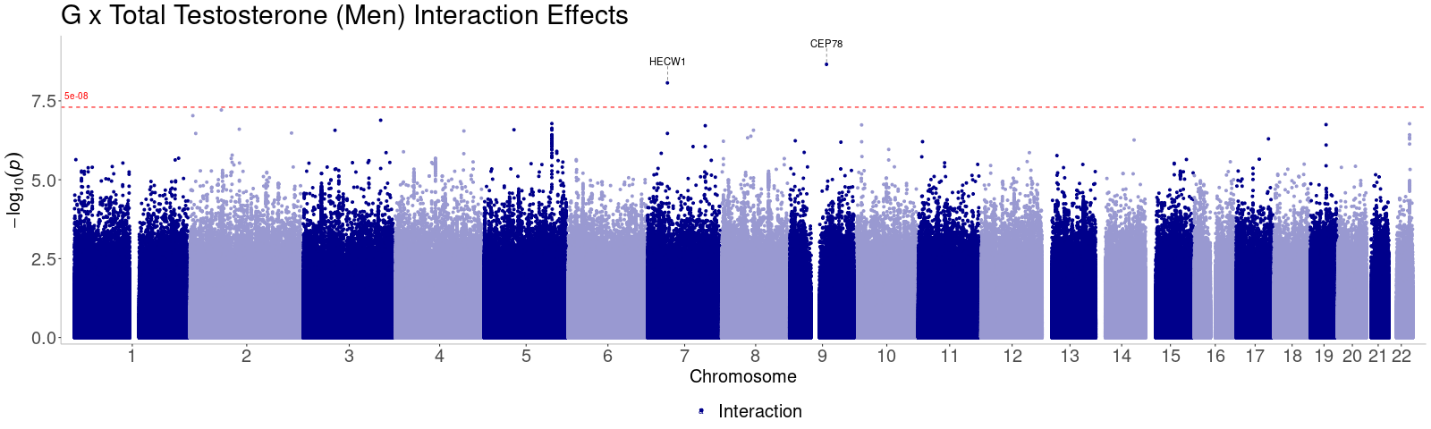
**

**B**

**
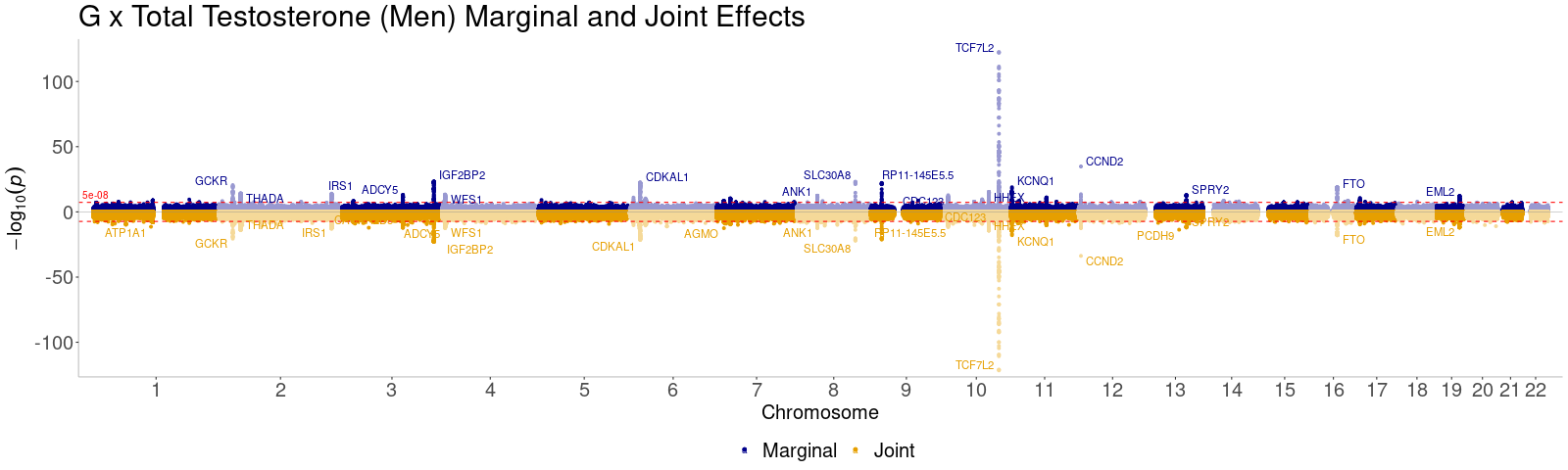
**

**C**


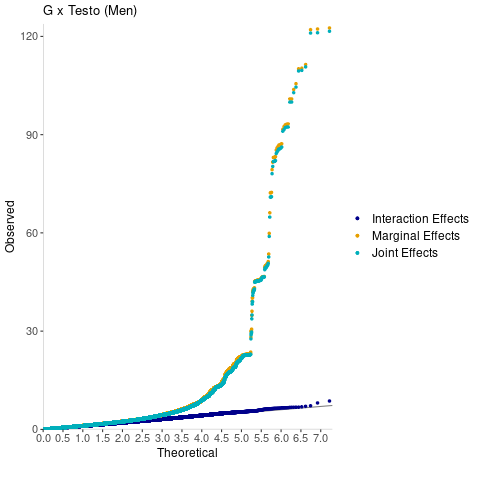


**S1.2 A-C Fig:** Manhattan and QQ Plots for the G x Total Testosterone analysis in women. GWAS inflation factors for interaction, joint, and marginal effects were 1.04, 1.13, and 1.14, respectively.

**A**


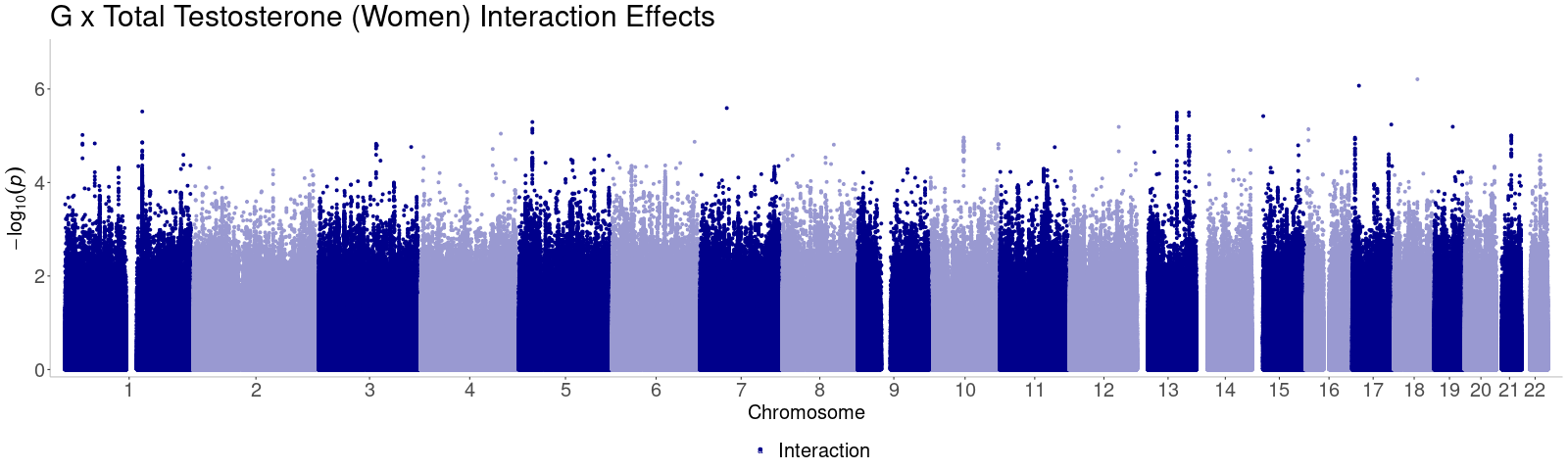


**B**
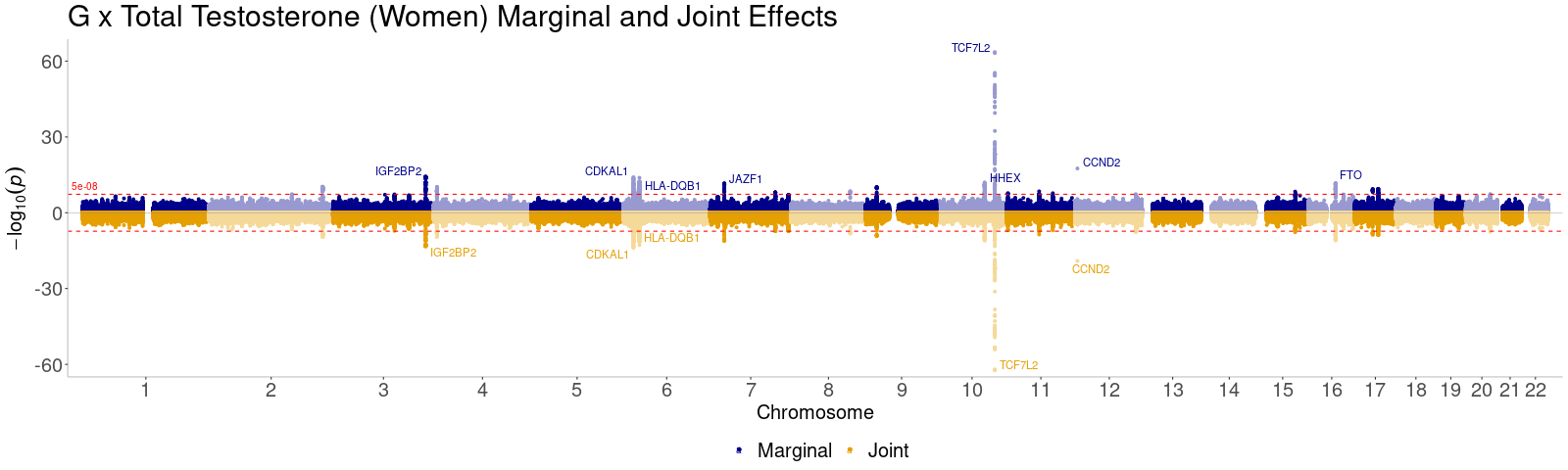


**C**


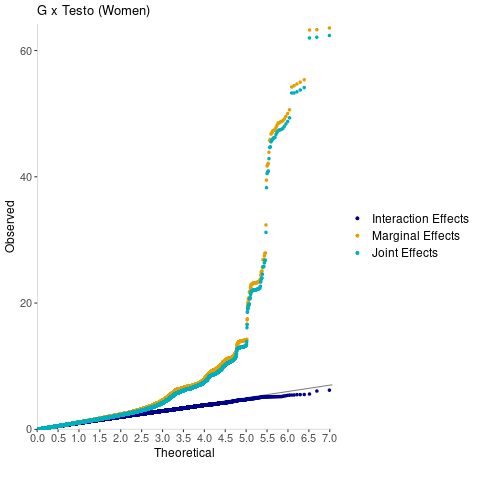


**S1.3 A-C Fig:** Manhattan and QQ Plots for the G x SHBG analysis in men. GWAS inflation factors for interaction, joint, and marginal effects were 1.01, 1.17, and 1.19, respectively.

**A**
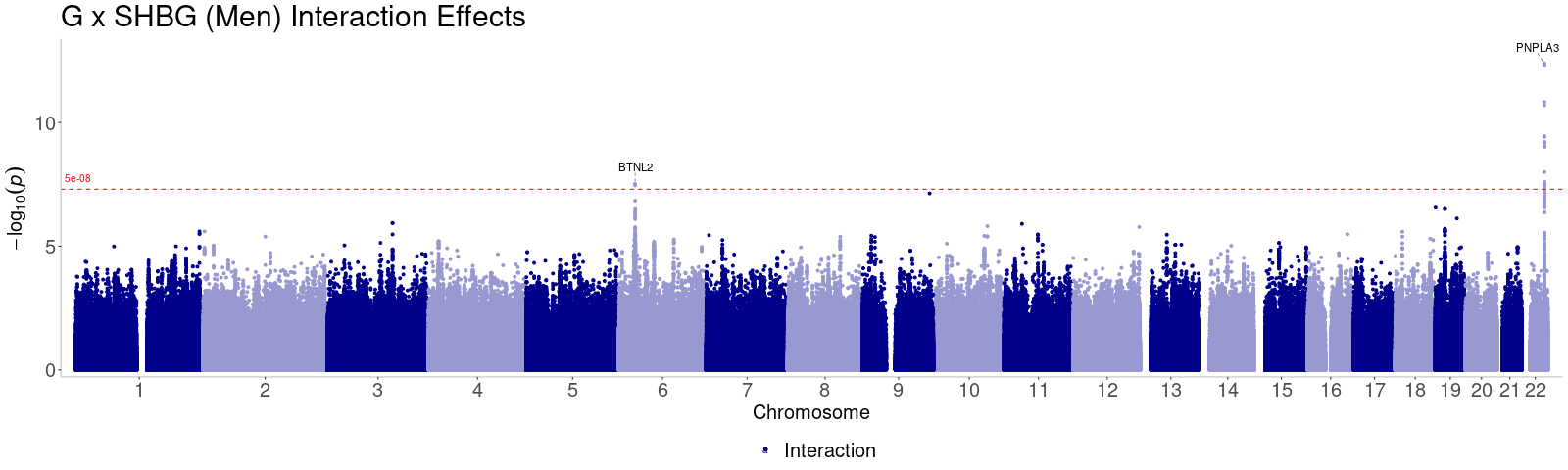


**B**
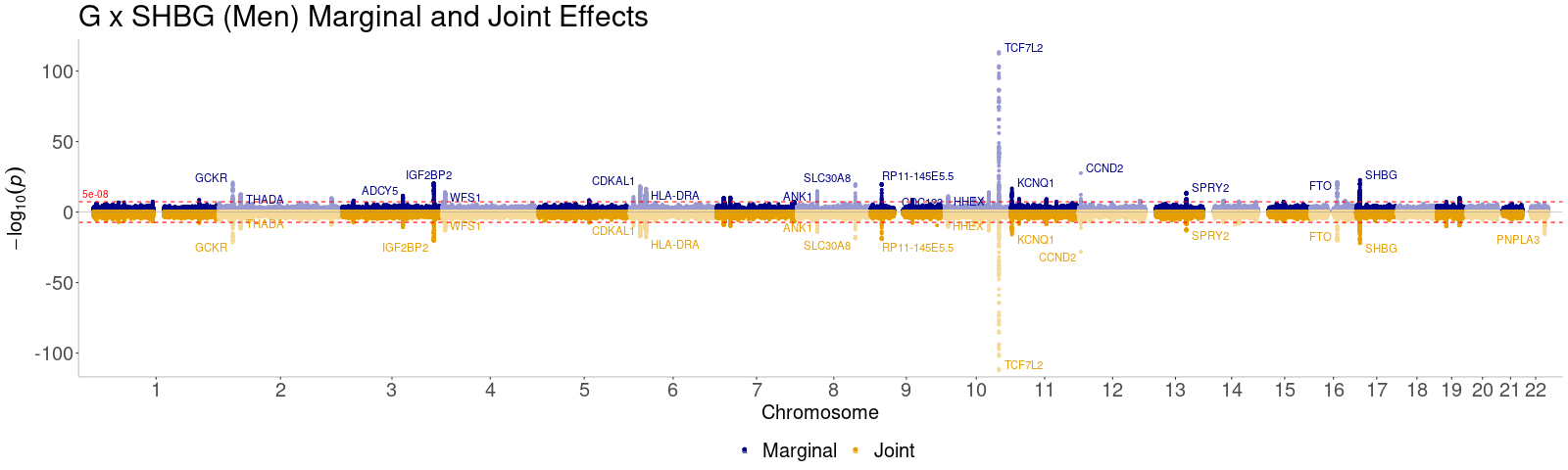


**C**


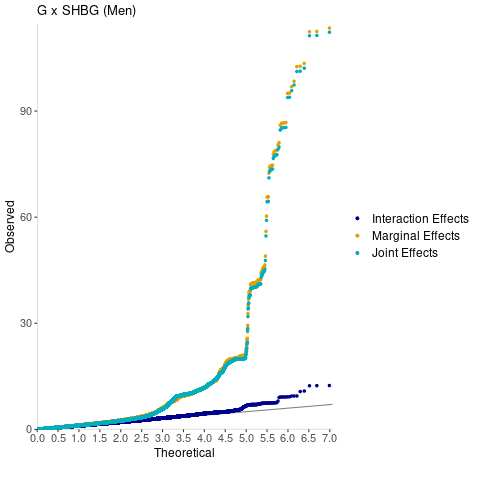


**S1.4 A-C Fig:** Manhattan and QQ Plots for the G x SHBG analysis in women. GWAS inflation factors for interaction, joint, and marginal effects were 1.03, 1.12, and 1.12, respectively.

**A**
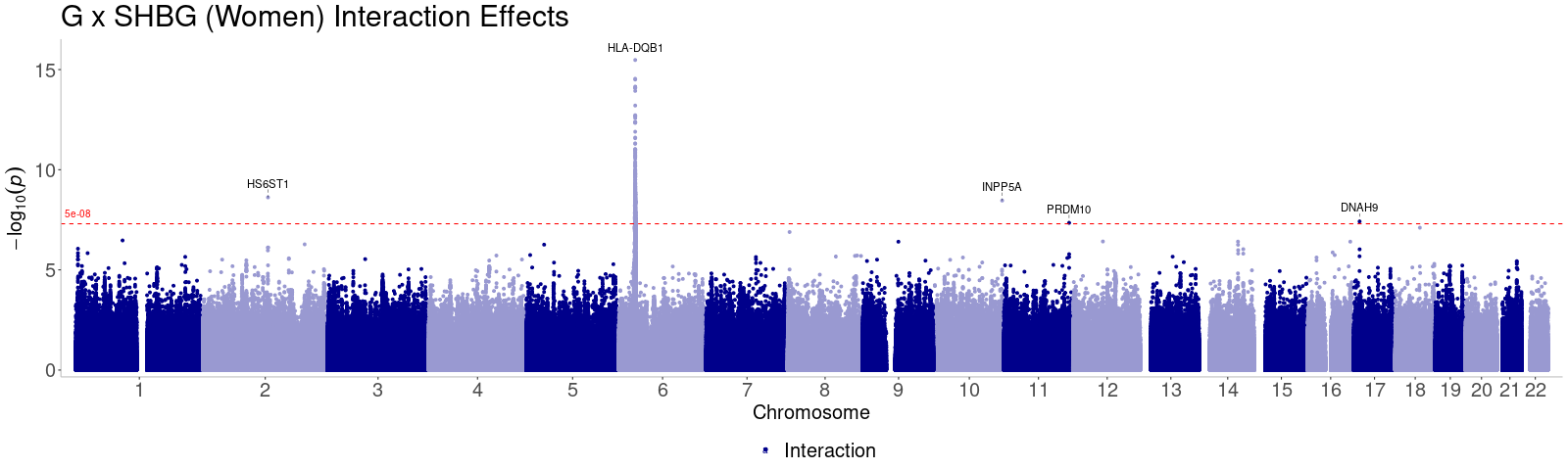


**B**
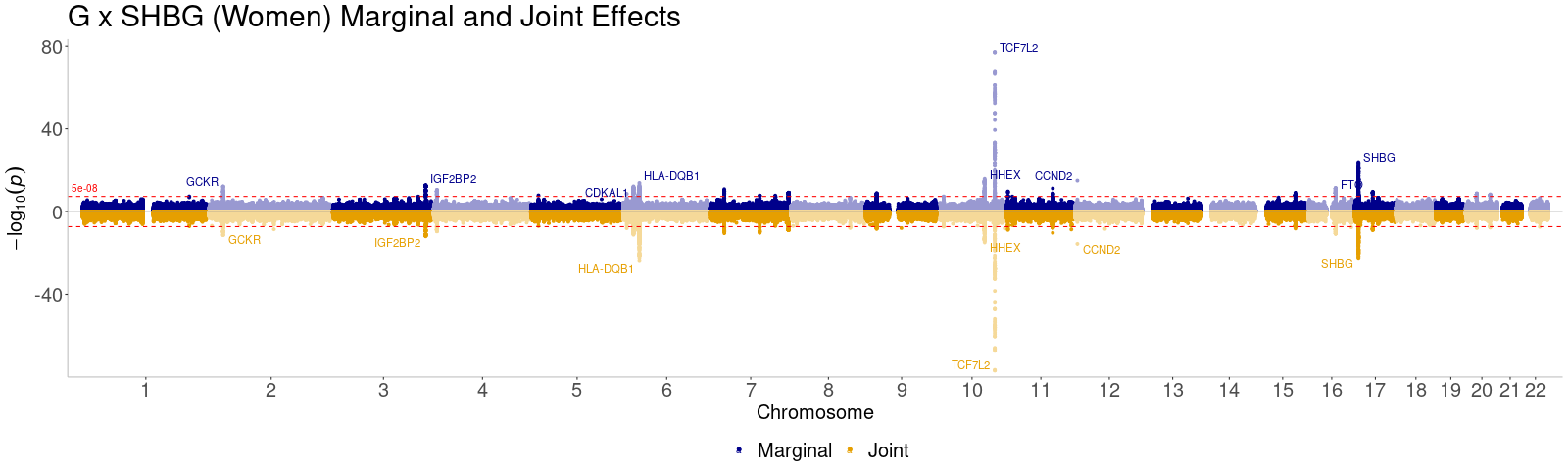


**C**


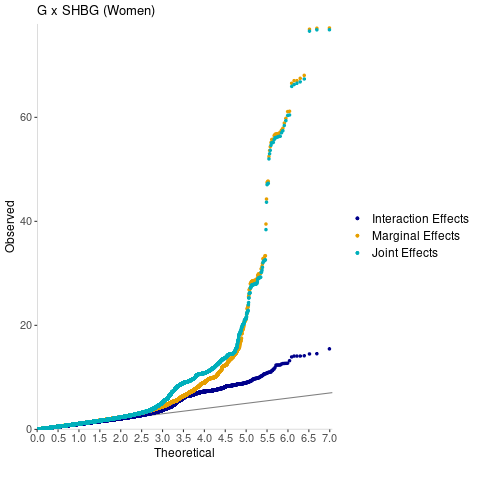


**S1.5 A-C Fig:** Manhattan and QQ Plots for the G x Bioavailable Testosterone analysis in men. GWAS inflation factors for interaction, joint, and marginal effects were 1.01, 1.16, and 1.18, respectively.

**A**
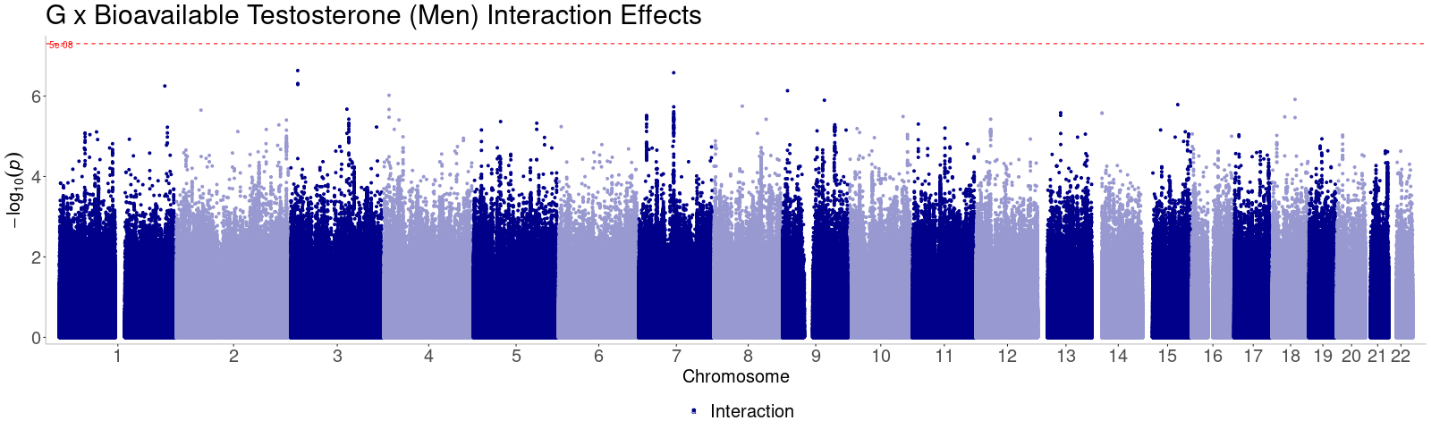


**B**
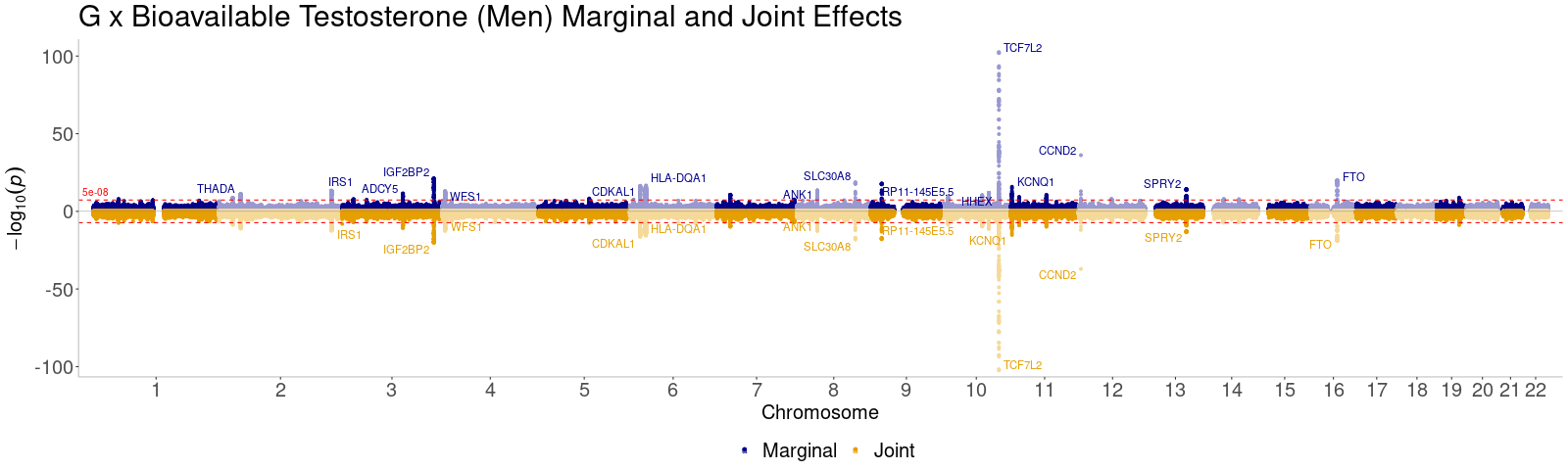


**C**


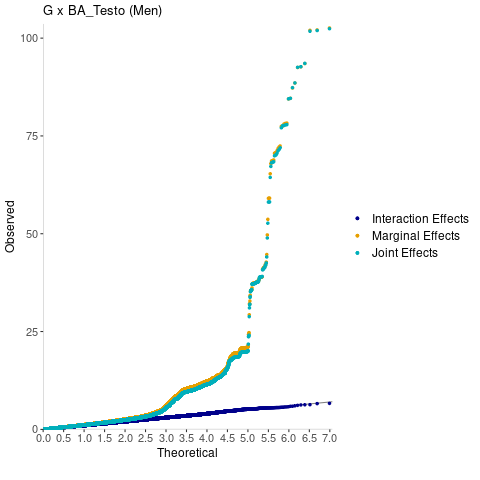


**S1.6 A-C Fig:** Manhattan and QQ Plots for the G x Bioavailable Testosterone analysis in women. GWAS inflation factors for interaction, joint, and marginal effects were 1.06, 1.14, and 1.11, respectively.

**A
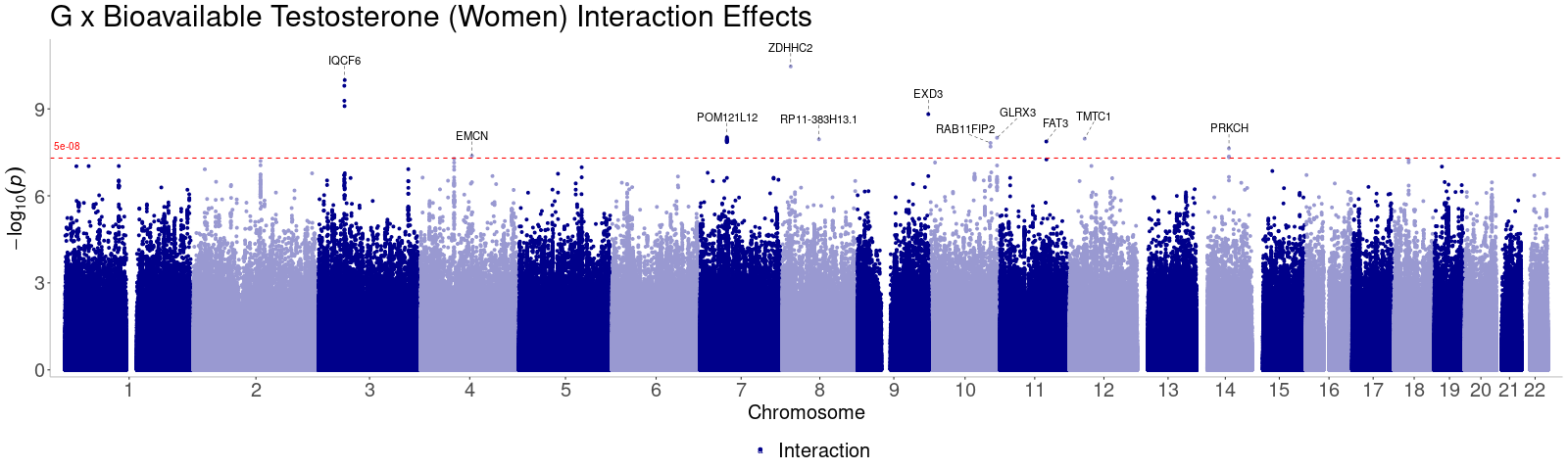
**

**B
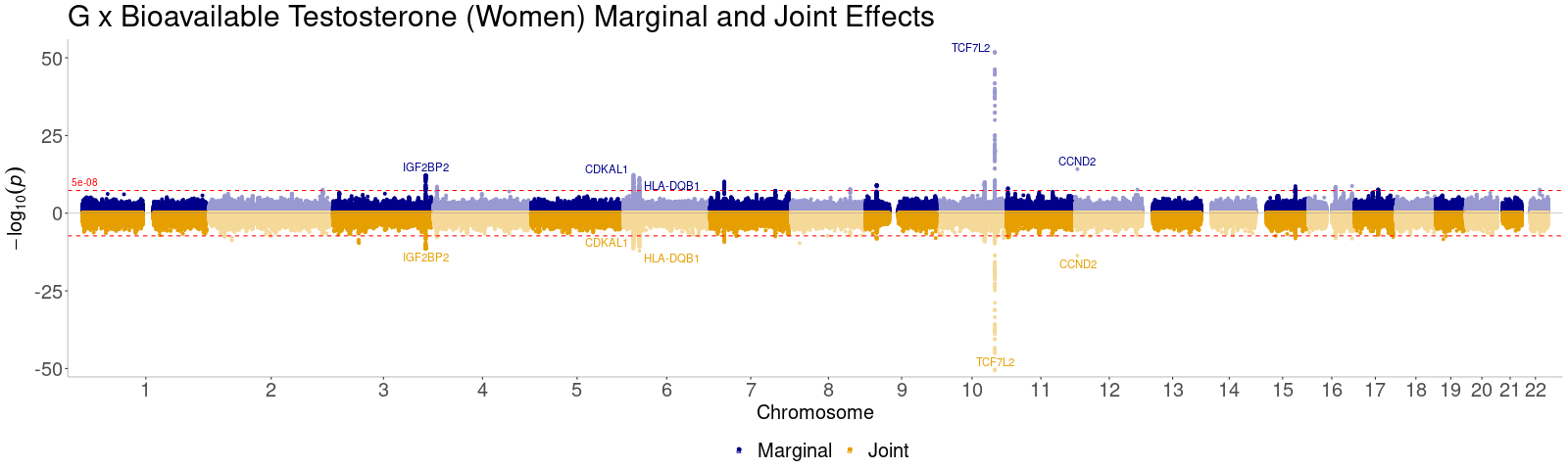
**

**C**


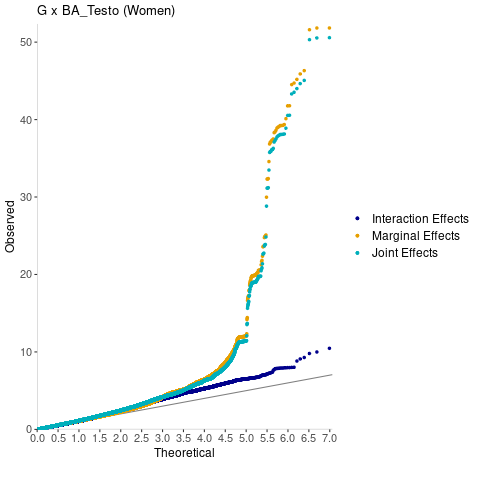

Supplement: S1 Fig — S1.2 A-C Fig. Manhattan and QQ Plots for the G x Total Testosterone analysis in women. S1.3 A-C Fig. Manhattan and QQ Plots for the G x SHBG analysis in men. S1.4 A-C Fig. Manhattan and QQ Plots for the G x SHBG analysis in women. S1.5 A-C Fig. Manhattan and QQ Plots for the G x Bioavailable Testosterone analysis in men. S1.6 A-C Fig. Manhattan and QQ Plots for the G x Bioavailable Testosterone analysis in women. (DOCX) [file pgen.1011470.s006.docx]
