# Supplementary material for: Identification of gene-sex hormone interactions associated with type 2 diabetes among men and women
Source: PLoS Genet. 2025 Sep 2;21(9):e1011470. doi: 10.1371/journal.pgen.1011470 (PMC12419643; doi:10.1371/journal.pgen.1011470)
Supplement: S2 Fig — (DOCX) [file pgen.1011470.s007.docx]

**S2 Fig**: Correlation plots for the effects of adding menopause status as a covariate on GWS SNPs in BAT (A) and SHBG (B) interaction (1 df) analyses.

**A**


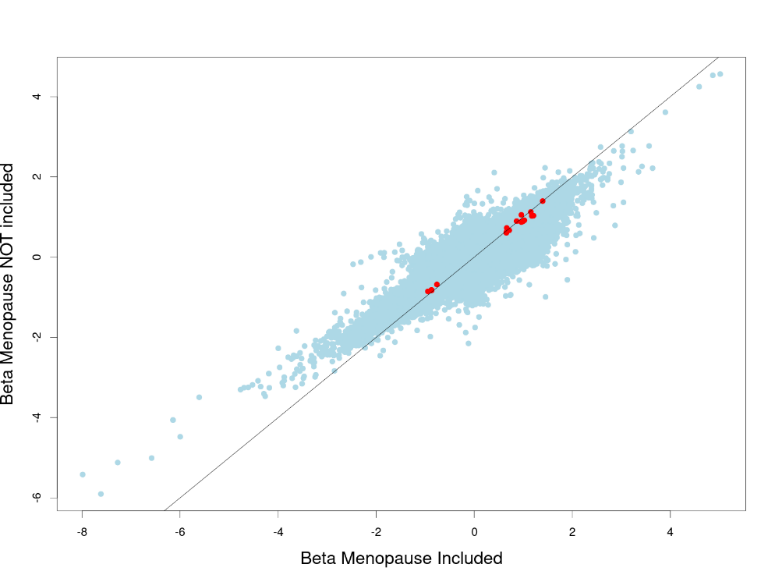


**B**


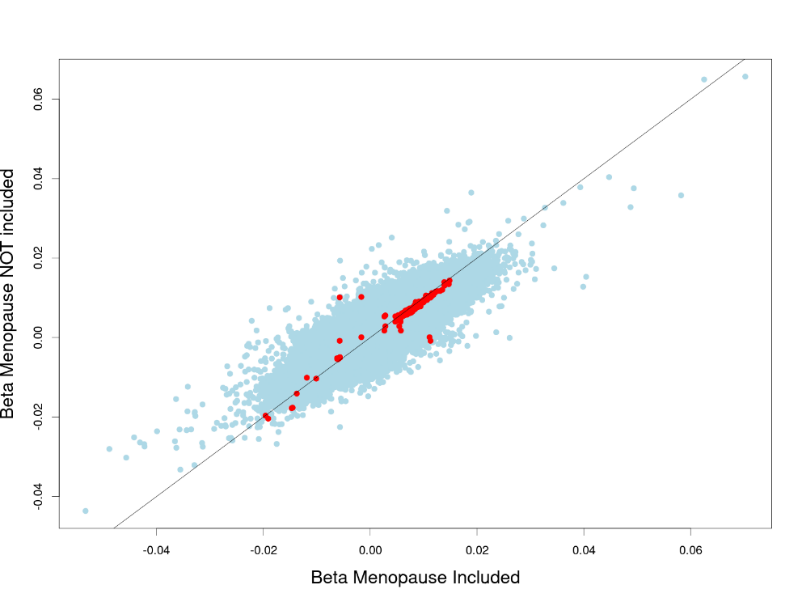


The correlation between interaction effect beta coefficients in GEM models with and without menopause as a covariate for G x BAT (A) and SHBG (B) analysis. “Beta Menopause NOT included” refers to the model with age at enrollment and PC1-10 as covariates and BAT or SHBG as the interaction term. “Beta Menopause Included” refers to the model with age at enrollment and PC1-10, and menopause as covariates and BAT or SHBG as the interaction term. Genome-wide significant SNPs in the original model without menopause are highlighted in red, and the line y = x is included for reference. For BAT, the correlation of all SNPs was 0.930, and the correlation of the 25 GWS SNPs was 0.998. For SHBG, the correlation of all SNPs was 0.859, and the correlation of the 947 GWS SNPs was 0.981. Abbreviations: SNP = single nucleotide polymorphism, BAT = bioavailable testosterone, SHBG = sex hormone binding globulin, PC = principal component, GWS = genome-wide significant.
