# Supplementary material for: Identification of gene-sex hormone interactions associated with type 2 diabetes among men and women
Source: PLoS Genet. 2025 Sep 2;21(9):e1011470. doi: 10.1371/journal.pgen.1011470 (PMC12419643; doi:10.1371/journal.pgen.1011470)
Supplement: S3 Fig — (DOCX) [file pgen.1011470.s008.docx]

**S3 Fig**: Correlation plots for the effects of excluding pre-menopausal women on GWS SNPs in BAT (A) and SHBG (B) interaction (1 df) analyses.

**A**


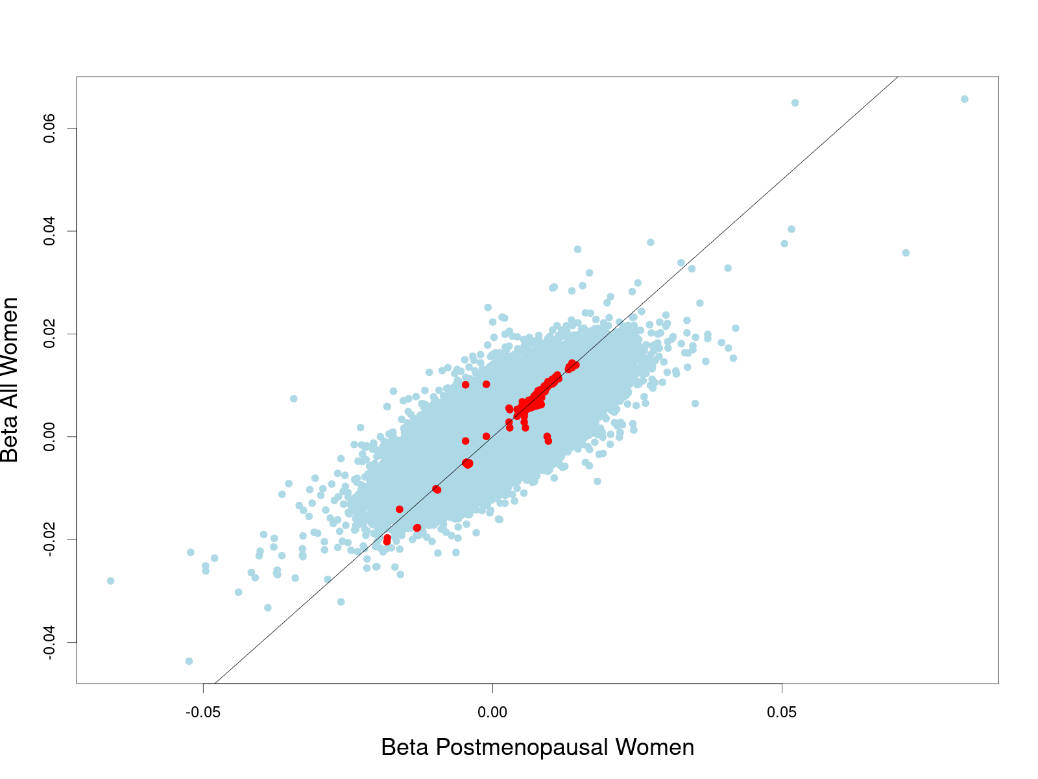


**B**


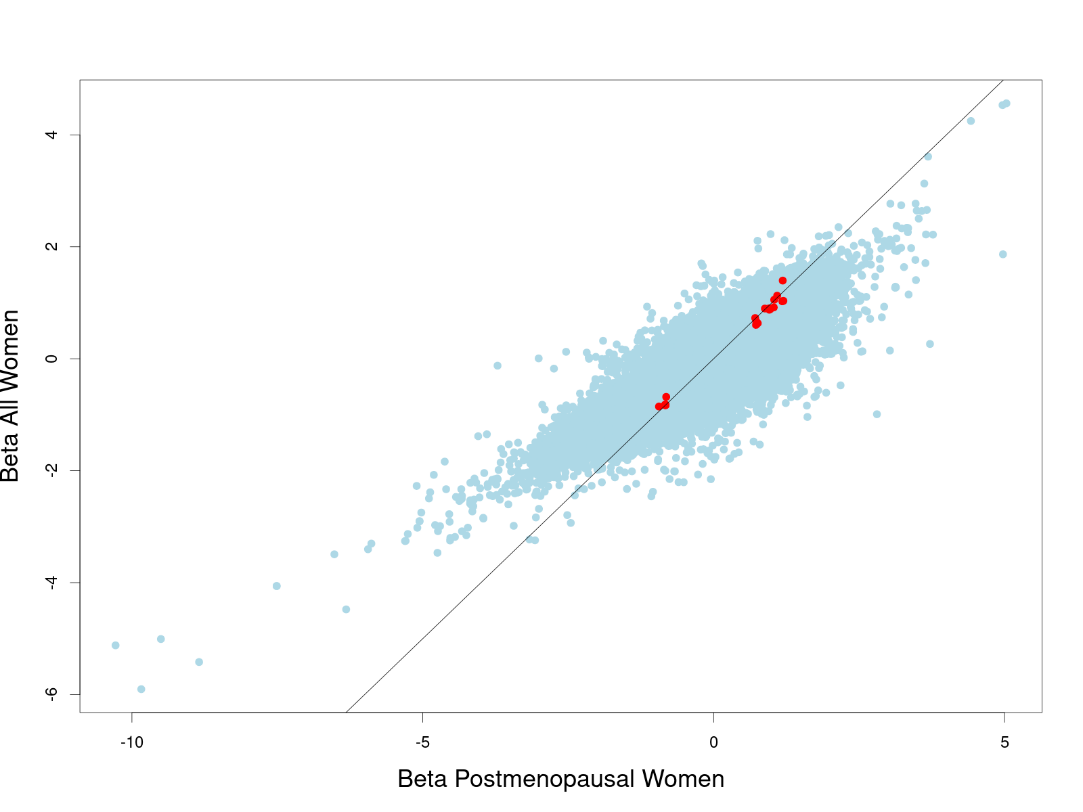


The correlation between interaction effect beta coefficients in GEM models in all women vs post-menopausal women for G x SHBG (A) and BAT (B) analysis. Genome-wide significant SNPs in the original model with all women included are highlighted in red, and the line y = x is included for reference. For SHBG, the correlation of all SNPs was 0.757, and the correlation of the 947 GWS SNPs was 0.979. For BAT, the correlation of all SNPs was 0.839, and the correlation of the 25 GWS SNPs was 0.996. Abbreviations: SNP = single nucleotide polymorphism, SHBG = sex hormone binding globulin, BAT = bioavailable testosterone, PC = principal component, GWS = genome-wide significant.
