# Supplementary material for: Identification of gene-sex hormone interactions associated with type 2 diabetes among men and women
Source: PLoS Genet. 2025 Sep 2;21(9):e1011470. doi: 10.1371/journal.pgen.1011470 (PMC12419643; doi:10.1371/journal.pgen.1011470)
Supplement: S4 Fig — (DOCX) [file pgen.1011470.s009.docx]

**S4 A-F Fig**: Correlation plots for incident type 2 diabetes sensitivity analyses.


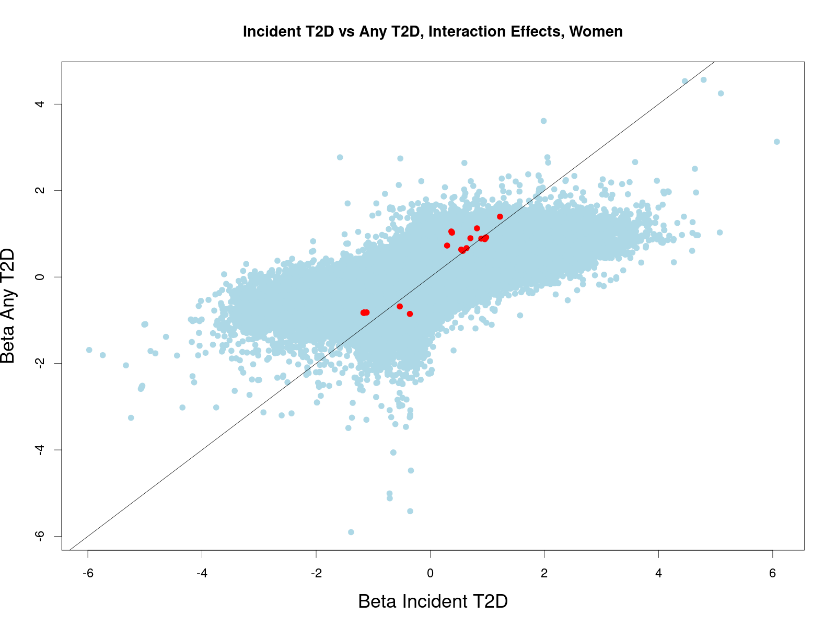

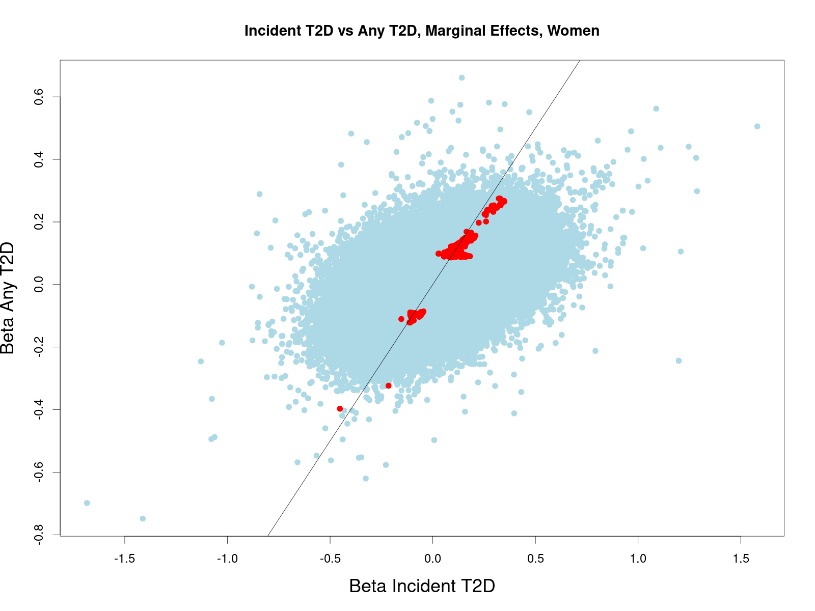


S4 A Fig: The correlation between marginal and interaction effect beta coefficients in G x BAT models with incident type 2 diabetes versus any type 2 diabetes (incident or prevalent) as an outcome in women. Age at enrollment and PC1-10 were covariates and BAT was the interaction term for both models. The model with incident type 2 diabetes as the outcome is represented on the x axis while the model with any type 2 diabetes is on the y axis. Genome-wide significant SNPs in the original model with any type 2 diabetes as the outcome are highlighted in red, and the line y = x is included for reference. The correlation of the 551 GWS SNPs (highlighted in red) associated with any type 2 diabetes identified using marginal effects is 0.956 while the correlation of all SNPs in the same analysis is 0.441. The correlation of the 25 GWS SNPs (highlighted in red) associated with any type 2 diabetes identified using interaction effects is 0.955 while the correlation of all SNPs in the same analysis is 0.641. Abbreviations: SNP = single nucleotide polymorphism, BAT = bioavailable testosterone, PC = principal component, GWS = genome-wide significant.


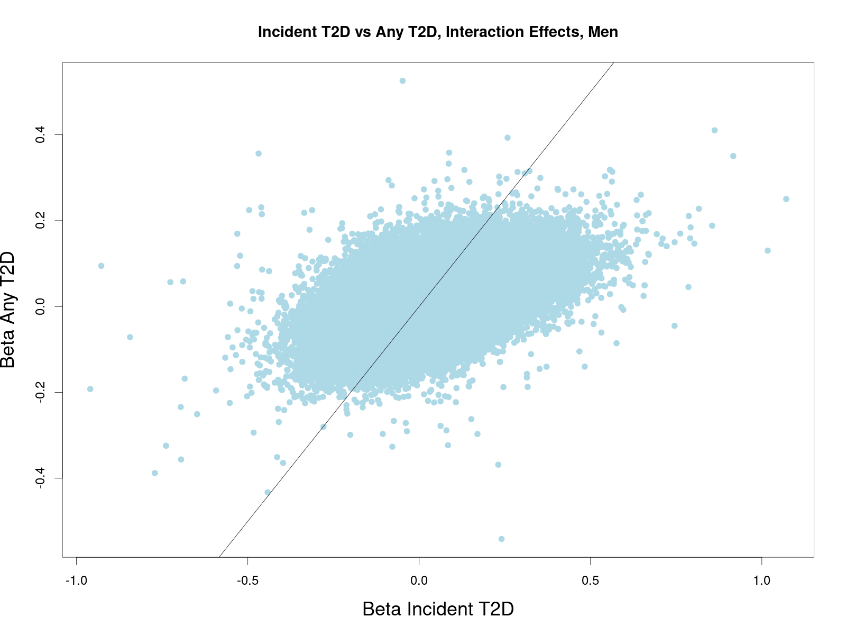

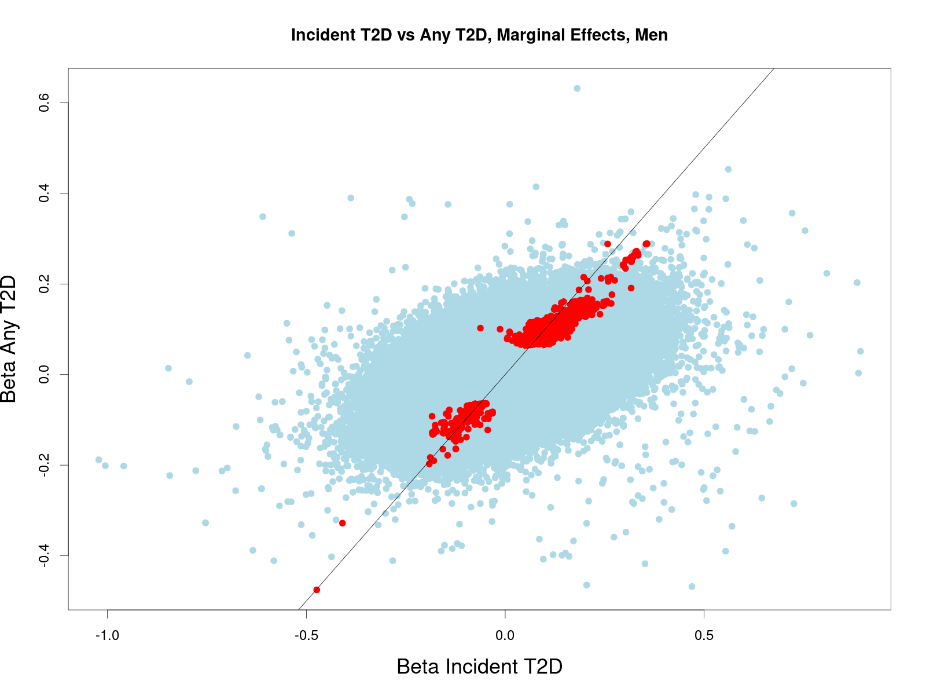


S4 B Fig: The correlation between marginal and interaction effect beta coefficients in G x BAT models with incident type 2 diabetes versus any type 2 diabetes (incident or prevalent) as an outcome in men. Age at enrollment and PC1-10 were covariates and BAT was the interaction term for both models. The model with incident type 2 diabetes as the outcome is represented on the x axis while the model with any type 2 diabetes is on the y axis. Genome-wide significant SNPs in the original model with any type 2 diabetes as the outcome are highlighted in red, and the line y = x is included for reference. The correlation of the 7445 GWS SNPs (highlighted in red) associated with any type 2 diabetes identified using marginal effects is 0.960 while the correlation of all SNPs in the same analysis is 0.507. The correlation of SNPs in the interaction analysis is 0.489. Abbreviations: SNP = single nucleotide polymorphism, BAT = bioavailable testosterone, PC = principal component, GWS = genome-wide significant.


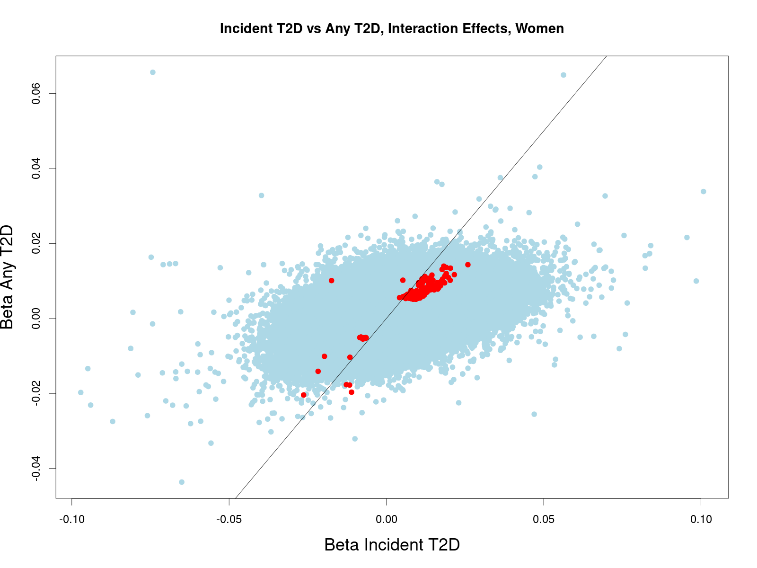

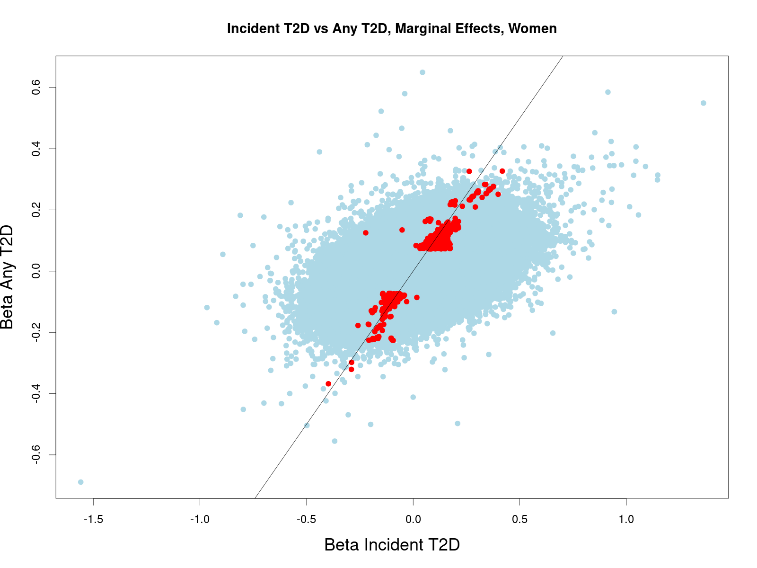


S4 C Fig: The correlation between marginal, joint and interaction effect beta coefficients in G x SHBG models with incident type 2 diabetes versus any type 2 diabetes (incident or prevalent) as an outcome in women. Age at enrollment and PC1-10 were covariates and SHBG was the interaction term for both models. The model with incident type 2 diabetes as the outcome is represented on the x axis while the model with any type 2 diabetes is on the y axis. Genome-wide significant SNPs in the original model with any type 2 diabetes as the outcome are highlighted in red, and the line y = x is included for reference. The correlation of the 1968 GWS SNPs (highlighted in red) associated with any type 2 diabetes identified using marginal effects is 0.963 while the correlation of all SNPs in the same analysis is 0.472. The correlation of the 953 GWS SNPs (highlighted in red) associated with any type 2 diabetes identified using interaction effects is 0.948 while the correlation of all SNPs in the same analysis is 0.440. Abbreviations: SNP = single nucleotide polymorphism, SHBG = sex hormone binding globulin, PC = principal component, GWS = genome-wide significant.


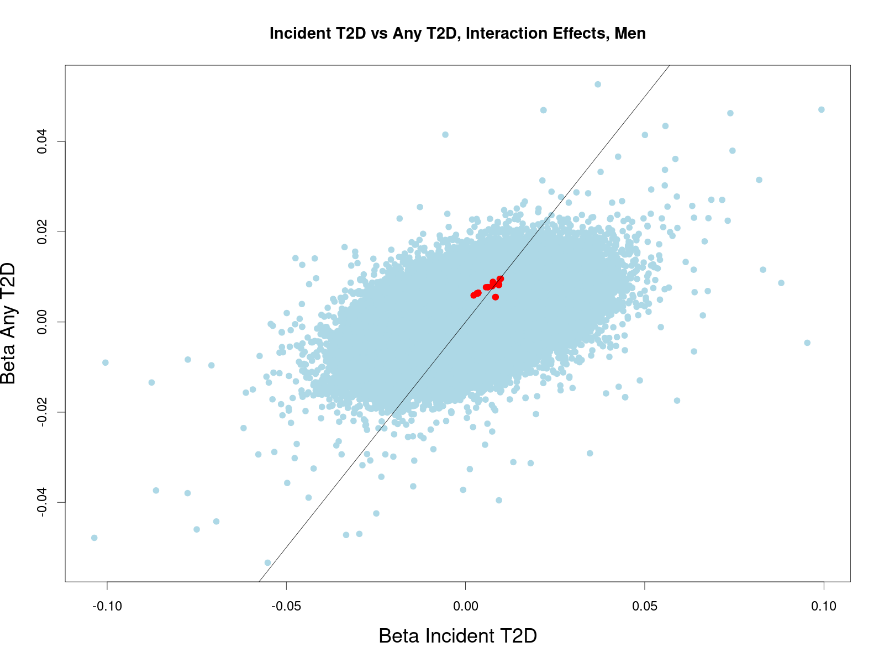

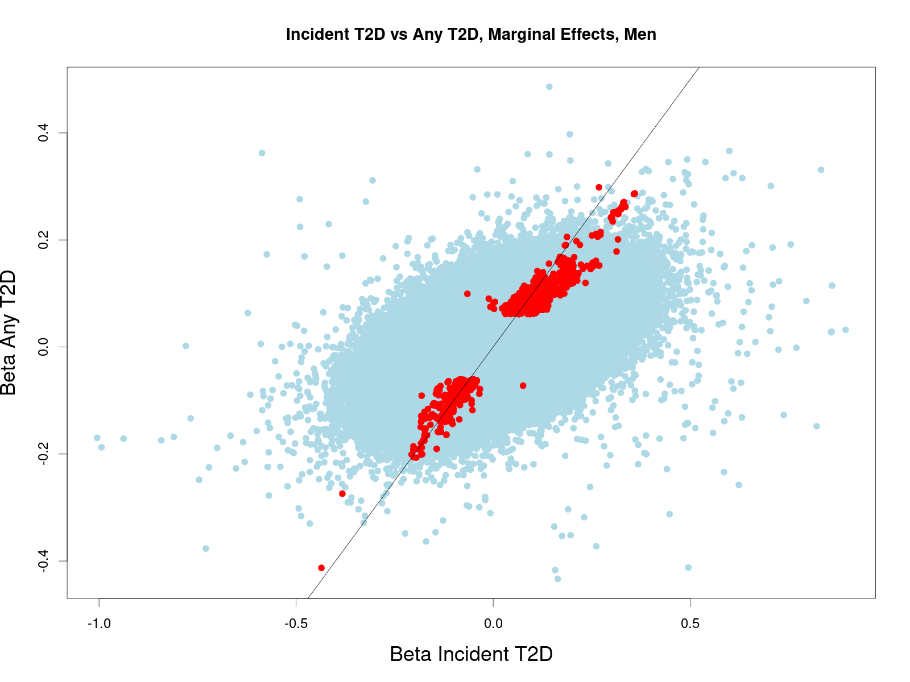


S4 D Fig: The correlation between marginal, joint and interaction effect beta coefficients in G x SHBG models with incident type 2 diabetes versus any type 2 diabetes (incident or prevalent) as an outcome in men. Age at enrollment and PC1-10 were covariates and SHBG was the interaction term for both models. The model with incident type 2 diabetes as the outcome is represented on the x axis while the model with any type 2 diabetes is on the y axis. Genome-wide significant SNPs in the original model with any type 2 diabetes as the outcome are highlighted in red, and the line y = x is included for reference. The correlation of the 7011 GWS SNPs (highlighted in red) associated with any type 2 diabetes identified using marginal effects is 0.959 while the correlation of all SNPs in the same analysis is 0.531. The correlation of the 42 GWS SNPs (highlighted in red) associated with any type 2 diabetes identified using interaction effects is 0.736 while the correlation of all SNPs in the same analysis is 0.511. Abbreviations: SNP = single nucleotide polymorphism, SHBG = sex hormone binding globulin, PC = principal component, GWS = genome-wide significant.


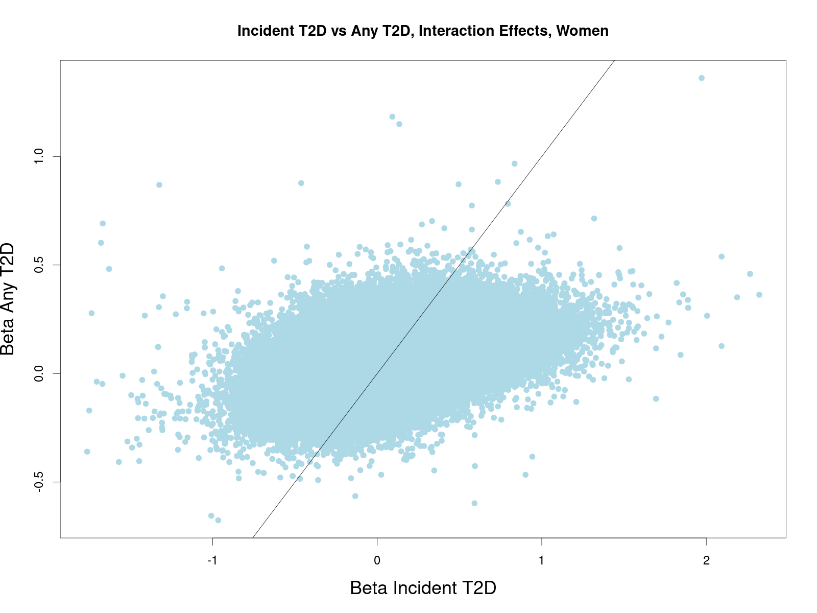

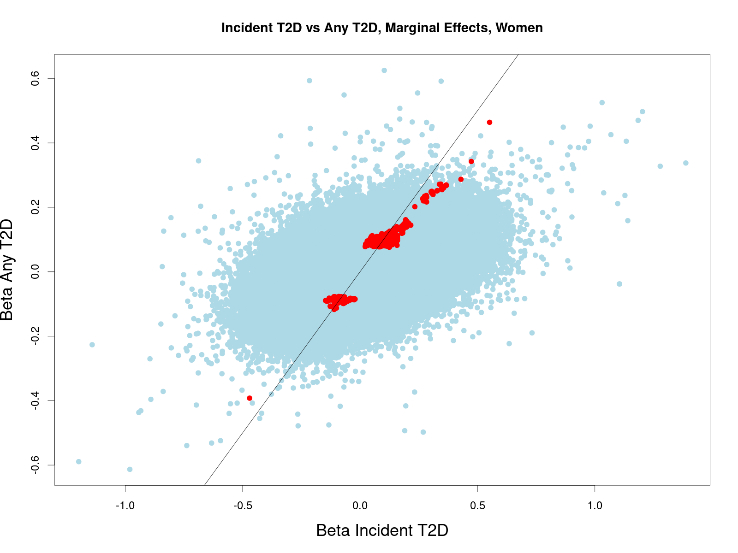


S4 E Fig: The correlation between marginal, joint and interaction effect beta coefficients in G x TT models with incident type 2 diabetes versus any type 2 diabetes (incident or prevalent) as an outcome in women. Age at enrollment and PC1-10 were covariates and TT was the interaction term for both models. The model with incident type 2 diabetes as the outcome is represented on the x axis while the model with any type 2 diabetes is on the y axis. Genome-wide significant SNPs in the original model with any type 2 diabetes as the outcome are highlighted in red, and the line y = x is included for reference. The correlation of the 1315 GWS SNPs (highlighted in red) associated with any type 2 diabetes identified using marginal effects is 0.940 while the correlation of all SNPs in the same analysis is 0.463. The correlation of SNPs in the interaction analysis is 0.410. Abbreviations: SNP = single nucleotide polymorphism, TT = total testosterone, PC = principal component, GWS = genome-wide significant.


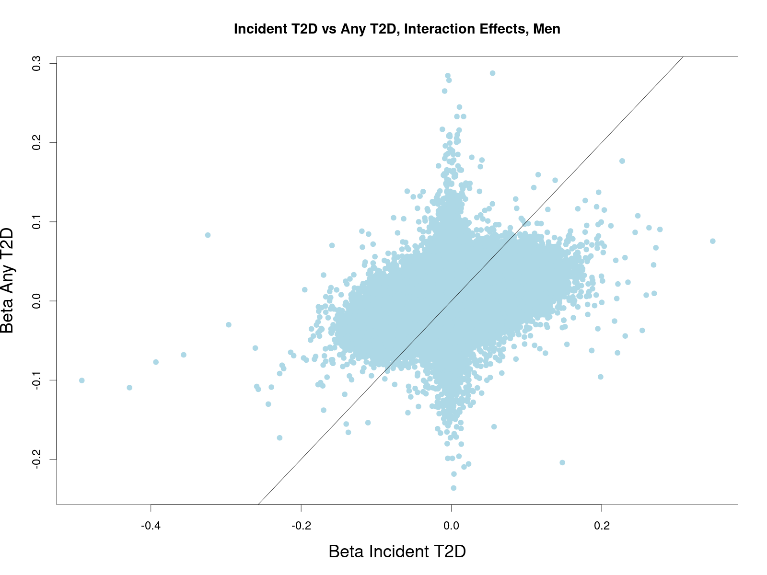

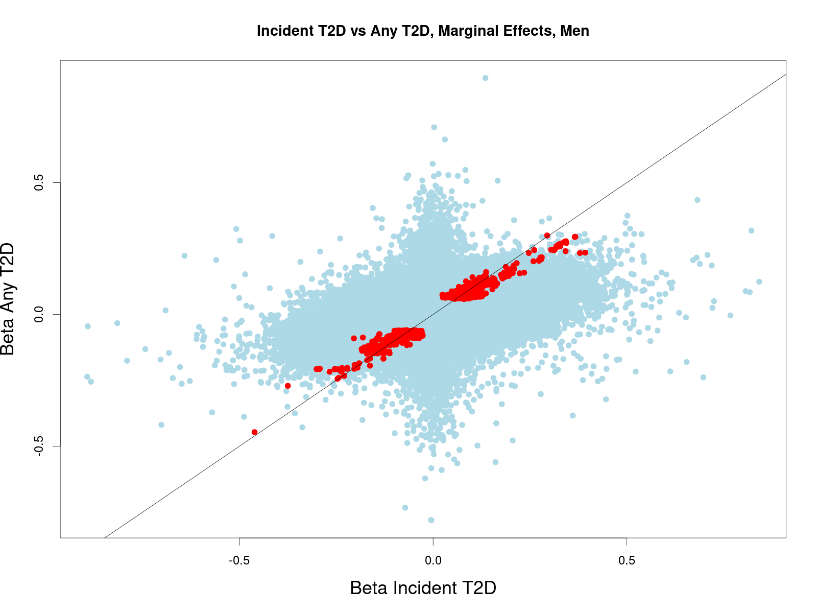


S4 F Fig: The correlation between marginal, joint and interaction effect beta coefficients in G x TT models with incident type 2 diabetes versus any type 2 diabetes (incident or prevalent) as an outcome in men. Age at enrollment and PC1-10 were covariates and TT was the interaction term for both models. The model with incident type 2 diabetes as the outcome is represented on the x axis while the model with any type 2 diabetes is on the y axis. Genome-wide significant SNPs in the original model with any type 2 diabetes as the outcome are highlighted in red, and the line y = x is included for reference. The correlation of the 2979 GWS SNPs (highlighted in red) associated with any type 2 diabetes identified using marginal effects is 0.985 while the correlation of all SNPs in the same analysis is 0.511. The correlation of SNPs in the interaction analysis is 0.472. Abbreviations: SNP = single nucleotide polymorphism, TT = total testosterone, PC = principal component, GWS = genome-wide significant.
