# Supplementary material for: Identification of gene-sex hormone interactions associated with type 2 diabetes among men and women
Source: PLoS Genet. 2025 Sep 2;21(9):e1011470. doi: 10.1371/journal.pgen.1011470 (PMC12419643; doi:10.1371/journal.pgen.1011470)
Supplement: S5 Table — (DOCX) [file pgen.1011470.s010.docx]

**S5 Table:** Summary statistics for the South Asian and African ancestry replication study cohorts.

| **South Asian** |  | **Female Cases** | **Male Cases** | **Female Controls** | **Male Controls** |
| --- | --- | --- | --- | --- | --- |
|  | **Count (%)** | 853 (11.37) | 1292 (17.22) | 2618 (34.90) | 2739 (36.51) |
|  | **Age (SD)** | 55.85 (7.41) | 56.64 (8.35) | 52.27 (8.14) | 52.22 (8.56) |
|  | **SHBG (SD)** | 37.53 (22.98) | 31.37 (14.55) | 52.42 (28.01) | 32.92 (14.42) |
|  | **TT (SD)** | 1.11 (0.63) | 10.51 (3.33) | 1.13 (0.60) | 11.71 (3.43) |
|  | **Albumin (SD)** | 43.96 (2.97) | 44.85 (2.92) | 44.13 (2.64) | 45.44 (2.67) |
|  | **PRS_T2D_ (SD)** | 0.47 (0.94) | 0.38 (0.94) | -0.11 (0.95) | -0.11 (0.95) |
| **African** |  |  |  |  |  |
|  | **Count (%)** | 836 (11.02) | 736 (9.70) | 3516 (46.34) | 2500 (32.95) |
|  | **Age (SD)** | 55.82 (8.04) | 55.88 (8.51) | 51.20 (7.62) | 50.62 (7.79) |
|  | **SHBG (SD)** | 44.13 (24.67) | 34.89 (15.65) | 60.37 (28.57) | 37.55 (15.97) |
|  | **TT (SD)** | 1.14 (0.60) | 11.10 (3.57) | 1.12 (0.58) | 12.79 (3.97) |
|  | **Albumin (SD)** | 43.73 (2.83) | 45.13 (2.99) | 43.94 (2.72) | 45.42 (2.71) |
|  | **PRS_T2D_ (SD)** | 0.42 (1.06) | 0.28 (1.09) | -0.03 (1.07) | -0.03 (1.08) |

*Data are presented as count (percentage) or mean (standard deviation) as appropriate. Cases refer to participants with type 2 diabetes; controls refer to participants with no diabetes. PRS_T2D_ – polygenic risk score for type 2 diabetes, TT – total testosterone, SHBG – sex hormone binding globulin, BAT – bioavailable testosterone*
