# Supplementary material for: Identification of gene-sex hormone interactions associated with type 2 diabetes among men and women
Source: PLoS Genet. 2025 Sep 2;21(9):e1011470. doi: 10.1371/journal.pgen.1011470 (PMC12419643; doi:10.1371/journal.pgen.1011470)
Supplement: S6 Table — (DOCX) [file pgen.1011470.s011.docx]

**S6 Table**: A comparison of genomic risk loci identified using interaction effects in Europeans to their counterparts in South Asian and African cohorts. The hormone column reflects the hormone used as an interaction term in the model. Dashes (-) represent analyses that could not be completed because the SNP fell below the minor allele frequency (0.01). Age at enrollment and PC1-10 were covariates in all models. Abbreviations: EU = European, ASN = South Asian, AFR = African, SE = standard error, BAT = bioavailable testosterone, SHBG = sex hormone binding globulin, TT = total testosterone, PC = Principal component.

| ***Men*** | | | | | | | | | | |
| --- | --- | --- | --- | --- | --- | --- | --- | --- | --- | --- |
| **SNPID** | **Hormone** | **Beta EU** | **SE EU** | **P EU** | **Beta ASN** | **SE ASN** | **P ASN** | **Beta AFR** | **SE AFR** | **P AFR** |
| rs738408 | SHBG | 0.01 | 0 | 4.09E-13 | -3.98E-03 | 5.18E-03 | 0.44 | -8.02E-03 | 7.89E-03 | 0.31 |
| rs3747207 | SHBG | 0.01 | 0 | 4.47E-13 | -3.93E-03 | 5.19E-03 | 0.45 | -6.41E-03 | 8.34E-03 | 0.44 |
| ***Women*** | | | | | | | | | | |
| **SNPID** | **Hormone** | **Beta EU** | **SE EU** | **P EU** | **Beta ASN** | **SE ASN** | **P ASN** | **Beta AFR** | **SE AFR** | **P AFR** |
| 8:17032214_CTT_C | BAT | -0.85 | 0.13 | 3.46E-11 | 0.35 | 0.83 | 0.68 | -0.65 | 0.4 | 0.1 |
| rs117878783 | BAT | 1.05 | 0.18 | 1.12E-08 | -0.56 | 1.33 | 0.68 | - | - | - |
| rs12268706 | BAT | 0.73 | 0.13 | 9.93E-09 | -0.63 | 0.86 | 0.47 | 0.17 | 0.64 | 0.79 |
| rs12825501 | BAT | 0.67 | 0.12 | 1.06E-08 | 0.06 | 1.07 | 0.95 | - | - | - |
| rs146037870 | BAT | 0.9 | 0.16 | 4.15E-08 | 0.29 | 1.77 | 0.87 | - | - | - |
| rs17256233 | BAT | 0.64 | 0.11 | 2.32E-08 | 0.21 | 0.43 | 0.62 | - | - | - |
| rs34789050 | BAT | 0.9 | 0.14 | 1.02E-10 | 0.87 | 1.05 | 0.4 | -0.28 | 1.08 | 0.8 |
| rs4476958 | BAT | -0.83 | 0.14 | 9.57E-09 | 0.21 | 0.51 | 0.68 | -0.21 | 0.91 | 0.82 |
| rs80123595 | BAT | 1.03 | 0.18 | 1.50E-08 | -0.34 | 1.64 | 0.84 | - | - | - |
| rs140069179 | SHBG | -0.01 | 0 | 3.83E-08 | -0.04 | 0.02 | 0.06 | - | - | - |
| rs9804606 | SHBG | -0.01 | 0 | 4.49E-08 | 0 | 0.02 | 0.87 | - | - | - |
