# Supplementary material for: Identification of gene-sex hormone interactions associated with type 2 diabetes among men and women
Source: PLoS Genet. 2025 Sep 2;21(9):e1011470. doi: 10.1371/journal.pgen.1011470 (PMC12419643; doi:10.1371/journal.pgen.1011470)
Supplement: S7 Table — (DOCX) [file pgen.1011470.s012.docx]

**S7 Table:** Expression quantitative trait loci (eQTL) identified through interaction effects. eQTLs were identified using the Genotype-Tissue Expression (GTEx) Project. P-Value refers to the statistical significance of the association between the SNP and gene expression in the tissue identified by the ‘Tissue’ column. NES – normalized effect size.

| **Gencode Id** | **Gene Symbol** | **Variant Id** | **SNP Id** | **P-Value** | **NES** | **Tissue** |
| --- | --- | --- | --- | --- | --- | --- |
| ENSG00000170322.15 | NFRKB | chr11_130019695_C_T_b38 | rs9804606 | 1.00E-07 | -0.5 | Adipose - Subcutaneous |
| ENSG00000170322.15 | NFRKB | chr11_130019695_C_T_b38 | rs9804606 | 0.00009 | -0.35 | Nerve - Tibial |
| ENSG00000170322.15 | NFRKB | chr11_130019695_C_T_b38 | rs9804606 | 0.00013 | -0.27 | Thyroid |
| ENSG00000164081.13 | TEX264 | chr3_51756013_G_A_b38 | rs34789050 | 2.60E-14 | 0.35 | Artery - Tibial |
| ENSG00000164081.13 | TEX264 | chr3_51756013_G_A_b38 | rs34789050 | 4.50E-10 | 0.38 | Esophagus - Muscularis |
| ENSG00000164082.15 | GRM2 | chr3_51756013_G_A_b38 | rs34789050 | 6.10E-10 | 0.32 | Muscle - Skeletal |
| ENSG00000164081.13 | TEX264 | chr3_51756013_G_A_b38 | rs34789050 | 1.20E-09 | 0.34 | Adipose - Subcutaneous |
| ENSG00000164082.15 | GRM2 | chr3_51756013_G_A_b38 | rs34789050 | 1.80E-09 | 0.31 | Skin - Sun Exposed (Lower leg) |
| ENSG00000164082.15 | GRM2 | chr3_51756013_G_A_b38 | rs34789050 | 5.60E-09 | 0.32 | Nerve - Tibial |
| ENSG00000164081.13 | TEX264 | chr3_51756013_G_A_b38 | rs34789050 | 5.70E-09 | 0.31 | Nerve - Tibial |
| ENSG00000164081.13 | TEX264 | chr3_51756013_G_A_b38 | rs34789050 | 9.20E-09 | 0.39 | Esophagus - Gastroesophageal Junction |
| ENSG00000164082.15 | GRM2 | chr3_51756013_G_A_b38 | rs34789050 | 1.60E-08 | 0.27 | Adipose - Subcutaneous |
| ENSG00000164081.13 | TEX264 | chr3_51756013_G_A_b38 | rs34789050 | 5.70E-08 | 0.23 | Cells - Cultured fibroblasts |
| ENSG00000145050.19 | MANF | chr3_51756013_G_A_b38 | rs34789050 | 8.60E-08 | 0.36 | Heart - Atrial Appendage |
| ENSG00000164081.13 | TEX264 | chr3_51756013_G_A_b38 | rs34789050 | 1.60E-07 | 0.21 | Muscle - Skeletal |
| ENSG00000164081.13 | TEX264 | chr3_51756013_G_A_b38 | rs34789050 | 2.30E-07 | 0.39 | Brain - Hippocampus |
| ENSG00000164082.15 | GRM2 | chr3_51756013_G_A_b38 | rs34789050 | 3.20E-07 | 0.5 | Brain - Nucleus accumbens (basal ganglia) |
| ENSG00000145050.19 | MANF | chr3_51756013_G_A_b38 | rs34789050 | 8.00E-07 | 0.39 | Testis |
| ENSG00000164081.13 | TEX264 | chr3_51756013_G_A_b38 | rs34789050 | 0.000001 | 0.48 | Vagina |
| ENSG00000164081.13 | TEX264 | chr3_51756013_G_A_b38 | rs34789050 | 1.1E-06 | 0.31 | Colon - Sigmoid |
| ENSG00000164082.15 | GRM2 | chr3_51756013_G_A_b38 | rs34789050 | 1.1E-06 | 0.31 | Testis |
| ENSG00000164081.13 | TEX264 | chr3_51756013_G_A_b38 | rs34789050 | 1.4E-06 | 0.2 | Thyroid |
| ENSG00000164081.13 | TEX264 | chr3_51756013_G_A_b38 | rs34789050 | 1.7E-06 | 0.25 | Adipose - Visceral (Omentum) |
| ENSG00000164081.13 | TEX264 | chr3_51756013_G_A_b38 | rs34789050 | 2.1E-06 | 0.27 | Breast - Mammary Tissue |
| ENSG00000164081.13 | TEX264 | chr3_51756013_G_A_b38 | rs34789050 | 3.3E-06 | 0.37 | Pancreas |
| ENSG00000164082.15 | GRM2 | chr3_51756013_G_A_b38 | rs34789050 | 4.9E-06 | 0.4 | Esophagus - Muscularis |
| ENSG00000164082.15 | GRM2 | chr3_51756013_G_A_b38 | rs34789050 | 6.4E-06 | 0.37 | Lung |
| ENSG00000164081.13 | TEX264 | chr3_51756013_G_A_b38 | rs34789050 | 7.5E-06 | 0.35 | Small Intestine - Terminal Ileum |
| ENSG00000164081.13 | TEX264 | chr3_51756013_G_A_b38 | rs34789050 | 0.000014 | 0.15 | Colon - Transverse |
| ENSG00000164081.13 | TEX264 | chr3_51756013_G_A_b38 | rs34789050 | 0.000016 | 0.29 | Artery - Aorta |
| ENSG00000164081.13 | TEX264 | chr3_51756013_G_A_b38 | rs34789050 | 0.000018 | 0.26 | Brain - Cerebellar Hemisphere |
| ENSG00000164082.15 | GRM2 | chr3_51756013_G_A_b38 | rs34789050 | 0.00005 | 0.33 | Breast - Mammary Tissue |
| ENSG00000164081.13 | TEX264 | chr3_51756013_G_A_b38 | rs34789050 | 0.000076 | 0.38 | Ovary |
| ENSG00000164081.13 | TEX264 | chr3_51756013_G_A_b38 | rs34789050 | 0.000082 | 0.27 | Brain - Nucleus accumbens (basal ganglia) |
| ENSG00000164081.13 | TEX264 | chr3_51756013_G_A_b38 | rs34789050 | 0.000084 | 0.18 | Esophagus - Mucosa |
| ENSG00000164081.13 | TEX264 | chr3_51756013_G_A_b38 | rs34789050 | 0.000097 | 0.11 | Whole Blood |
| ENSG00000145050.19 | MANF | chr3_51756013_G_A_b38 | rs34789050 | 0.00011 | 0.32 | Colon - Sigmoid |
| ENSG00000164082.15 | GRM2 | chr3_51756013_G_A_b38 | rs34789050 | 0.00017 | 0.25 | Whole Blood |
| ENSG00000164082.15 | GRM2 | chr3_51756013_G_A_b38 | rs34789050 | 0.00026 | 0.21 | Artery - Tibial |
| ENSG00000164082.15 | GRM2 | chr3_51756013_G_A_b38 | rs34789050 | 0.00036 | 0.36 | Brain - Caudate (basal ganglia) |
| ENSG00000164082.15 | GRM2 | chr3_51756013_G_A_b38 | rs34789050 | 0.00064 | 0.17 | Skin - Not Sun Exposed (Suprapubic) |
| ENSG00000165323.16 | FAT3 | chr11_92086422_A_G_b38 | rs117530301 | 0.00019 | 0.49 | Cells - Cultured fibroblasts |
| ENSG00000257599.4 | OVCH1-AS1 | chr12_29981626_T_C_b38 | rs12825501 | 0.00028 | -0.38 | Cells - Cultured fibroblasts |
|  |  |  |  |  |  |  |
